# Detailed Functional and Proteomic Characterization of Fludarabine Resistance in Mantle Cell Lymphoma Cells

**DOI:** 10.1371/journal.pone.0135314

**Published:** 2015-08-18

**Authors:** Lucie Lorkova, Michaela Scigelova, Tabiwang Ndipanquang Arrey, Ondrej Vit, Jana Pospisilova, Eliska Doktorova, Magdalena Klanova, Mahmudul Alam, Petra Vockova, Bokang Maswabi, Pavel Klener, Jiri Petrak

**Affiliations:** 1 Institute of Pathological Physiology, First Faculty of Medicine, Charles University in Prague, Prague, Czech Republic; 2 Thermo Fisher Scientific, Bremen, Germany; 3 First Department of Medicine—Department of Hematology, General University Hospital and Charles University in Prague, Prague, Czech Republic; 4 Institute of Hematology and Blood Transfusion, Prague, Czech Republic; University of Colorado, School of Medicine, UNITED STATES

## Abstract

Mantle cell lymphoma (MCL) is a chronically relapsing aggressive type of B-cell non-Hodgkin lymphoma considered incurable by currently used treatment approaches. Fludarabine is a purine analog clinically still widely used in the therapy of relapsed MCL. Molecular mechanisms of fludarabine resistance have not, however, been studied in the setting of MCL so far. We therefore derived fludarabine-resistant MCL cells (Mino/FR) and performed their detailed functional and proteomic characterization compared to the original fludarabine sensitive cells (Mino). We demonstrated that Mino/FR were highly cross-resistant to other antinucleosides (cytarabine, cladribine, gemcitabine) and to an inhibitor of Bruton tyrosine kinase (BTK) ibrutinib. Sensitivity to other types of anti-lymphoma agents was altered only mildly (methotrexate, doxorubicin, bortezomib) or remained unaffacted (cisplatin, bendamustine). The detailed proteomic analysis of Mino/FR compared to Mino cells unveiled over 300 differentially expressed proteins. Mino/FR were characterized by the marked downregulation of deoxycytidine kinase (dCK) and BTK (thus explaining the observed crossresistance to antinucleosides and ibrutinib), but also by the upregulation of several enzymes of de novo nucleotide synthesis, as well as the up-regulation of the numerous proteins of DNA repair and replication. The significant upregulation of the key antiapoptotic protein Bcl-2 in Mino/FR cells was associated with the markedly increased sensitivity of the fludarabine-resistant MCL cells to Bcl-2-specific inhibitor ABT199 compared to fludarabine-sensitive cells. Our data thus demonstrate that a detailed molecular analysis of drug-resistant tumor cells can indeed open a way to personalized therapy of resistant malignancies.

## Introduction

Mantle cell lymphoma (MCL) is a chronically relapsing aggressive type of B-cell non-Hodgkin lymphoma. Its estimated annual incidence in Europe is 0.45/100,000 persons. MCL remains incurable; despite the fact that most patients achieve an objective response (complete or partial remisison) after induction therapy, virtually all patients relapse sooner or later [1,2]. MCL is characterized by the translocation t(11;14)(q13;q32) leading to the overexpression of cyclin D1 with the ensuing deregulation of cell cycle machinery. Additional molecular aberrations include mutation in ATM, TP53, CDKN2A, RB1, CDK4/6, and MDM2, among other genes [1,3].

Newly diagnosed MCL patients are typically subjected to a combinatorial immunochemotherapy comprising anti-CD20 antibody rituximab (R), intensified anthracycline-based chemotherapy (e.g. R-Maxi-CHOP: cyclophosphamide, vincristine, doxorubicin, and prednisone), high-dose cytarabine (R-HDAC), and consolidation with high-dose therapy and autologous stem cell rescue. Prognosis of relapsed/refractory MCL is poor [1,2], no standard of care has been defined for such a condition. Second-line treatment approaches are based on nucleoside analogs (fludarabine, cladribine), DNA modifying agents (bendamustine, cisplatin), or targeted therapeuticals (bortezomib, temsirolimus, lenalidomide or ibrutinib). In everyday clinical practice, fludarabine-based regimens still remain important and widely used options for the salvage therapy of relapsed/refractory MCL [4]. In addition, several novel multi-agent combinations incorporating fludarabine have been tested and showed promise in the therapy of RR-MCL [5]. Outside MCL, fludarabine-based regimens are used for the first-line therapy of chronic lymphocytic leukemia (CLL), and the salvage therapy of indolent lymphomas and acute myelogeneous leukemias (AML).

Fludarabine (9-beta-d-arabinofuranosyl-2-fluoroadenine) is a prodrug administered in the form of a monophosphate (F-ara-AMP) which is dephosphorylated in vivo by plasma phosphatases and transported into cells. There it is retained after its re-phosphorylation to monophospate by deoxycytidine kinase (dCK) in the rate limiting step of fludarabine utilization. Next, F-ara-AMP is phosphorylated by adenylate kinase to a diphosphate F-ara-ADP, and by nucleoside diphosphate kinase to a triphosphate F-ara-ATP representing the active form of the drug. Incorporation of F-ara-ATP into DNA results in chain termination, replication fork stalling, and DNA breaks [6,7]. The replication stress activates DNA damage response resulting in either DNA repair or apoptosis. In addition, F-ara-ATP also decreases available dNTP pool by inhibiting ribonucleotide reductase, an enzyme critical for sufficient supply of deoxyribonucleotides. Fludarabine is also incorporated into RNA, and has been shown to directly induce apoptosis via caspase activation [6].

Unfortunately, acquired resistance to fludarabine is frequent. Mechanisms of fludarabine resistance in lymphomas are largely unknown. So far, several studies reported various molecules mutated or deregulated in association with fludarabine refractoriness (i.e. inherent resistance or acquired resistance in leukemic cells (CLL, AML)), including molecules/genes involved in the nucleotide salvage pathway (dCK, nucleotide transporters, ribonucleotide reductase) [8–11], antiapoptotic molecules (BCL2 family, BIRC3), transcription factors (MYC, NOTCH), mediators of genotoxic stress (ATM, TP53, SF3B1) and others (SULF2, MTORC2) [12–20]. Molecular mechanisms of fludarabine resistance thus appear highly heterogeneous, and potentially might even be disease-specific, a consequence of different genetic (mutational) background between acute myeloid and chronic lymphoid leukemias. To the best of our knowledge, the molecular mechanisms of fludarabine resistance in MCL have not been studied so far.

In this manuscript we derived fludarabine-resistant MCL cells, and studied their sensitivity to clinically-approved and experimental anti-lymphoma agents in order to empirically identify optimal strategies for elimination of fludarabine-resistant MCL cells. In addition, using detailed SILAC-based proteomic analysis we described molecular events responsible for and associated with fludarabine resistance in MCL, including causative, contributing and adaptive cellular processes.

## Materials and Methods

### Establishment of fludarabine-resistant clones

MCL cell line Mino [21] was purchased from ATCC, and cultured in Iscove’s modified Dulbecco’s medium (IMDM) supplemented with 15% fetal bovine serum (FBS) and 1% penicillin/streptomycin under standard tissue culture conditions. Fludarabine-resistant Mino cells (Mino/FR) were derived by co-culture with gradually increasing doses of fludarabine up to 100 μM.

### Proliferation assays

Cytarabine, fludarabine, gemcitabine, cladribine, doxorubicin and cisplatin were from Clinical Dept. of Hematology, University Hospital in Prague, Czech Republic. Bortezomib, bendamustine, ABT-199 and ibrutinib were purchased from Selleck Chemicals. Proliferation was estimated using WST-8 Quick Cell Proliferation Assay Kit (BioVision) according to the manufacturer instructions. Briefly, 10,000 cells were seeded into a 96-well plate on day 1. Drugs were added on day 1. Proliferation was measured on day 1, and then starting on day 4 on a daily basis. The antiproliferative activity of each drug was analyzed at several concentrations. Absorbance of the samples (triplicate) was measured on ELISA reader after a three-hour incubation with WST-8 reagent at 37°C in a thermostat. Maximum absorbance (MAXu) obtained from the untreated cells during the particular experiment was arbitrarily set to represent 100%. Absorbance of medium without cells was used as background (B). For each cell population (i.e. unexposed and drug-exposed) and for each measurement (M1, M2, M3…MX) the proliferation curve was calculated as follows: (MX—B)/(MAXu—B). As a consequence, the proliferation curve of untreated cells always peaks at 100%, while proliferation curves of drug-exposed cells can terminate below or above 100%.

### SILAC labeling

Mino cells were maintained in DMEM medium. To prepare SILAC heavy and light media, SILAC DMEM (Arg, Lys and Leu free) was supplemented with 10% dialyzed fetal bovine serum (FBS), 1% penicillin-streptomycin, sodium pyruvate (1 mM), glucose (4.5 g/L). The light (L) and heavy (H) media were supplemented with either 146 mg/L of L-lysine (L) or L-[^13^C_6_, ^15^N_2_] lysine (H) and either 84 mg/L of L-arginine (L) or L-[^13^C_6_, ^15^N_4_] arginine (H). L-proline (200 mg/L, Thermo Scientific) was added to both media to avoid the conversion of arginine to proline. All other chemicals were obtained from Sigma-Aldrich (St. Louis, MO, USA).

Mino cells were first grown in the “heavy media” (forward labeling) and Mino/FR in the “light media”. In a parallel experiment, the media were swapped (reverse labeling). The cells were grown for at least six generations, and the complete incorporation of heavy amino acids was verified by a mass spectrometric analysis.

The same amounts (10 x 10^6^ cells) of “light” and “heavy” labeled cells were mixed and processed further. The cells were washed three times with PBS. The mixed cell pellets (20 x 10^6^ cells) were homogenized in 160 μL lysis buffer (10 mM HEPES, pH 7.4, 140 mM NaCl, 1.5% Triton X-100) at 4°C for 10 min. The whole cell lysates were centrifuged for 10 min (18,000 g) at 4°C. Protein concentration was determined using Bradford assay (Bio-Rad, CA, USA).

### Filter-aided sample preparation (FASP)

Whole cell lysates (H+L) were digested using the filter-aided sample preparation (FASP) method [22] enabling detergent removal, reduction, alkylation and digestion on a filter. Cell lysates (100 μg total protein) were mixed with 300 μL of 8 M urea in 0.1 M Tris/HCl, pH 8.5 (UA buffer) supplemented with 100 mM DTT and incubated for 15 min at room temperature. After centrifugation at 18,000 g for 10 min, the supernatant was loaded on Ultrafree-MC centrifugal filter with a nominal molecular weight cutoff of 10,000 Da (Sigma-Aldrich, St. Louis, MO, USA) and centrifuged at 5,000 g. Retenate was diluted again in 300 μL of UA buffer and centrifuged until the complete removal of UA. The proteins were then alkylated with 100 μL 50 mM iodoacetamide dissolved in UA, incubated for 20 min at room temperature in the dark. Samples were then washed twice with 100 μL UA and three times with 100 μL of 50 mM ammonium bicarbonate. Proteins were digested with trypsin in the filter cone in 40 μL of 50 mM ammoniom bicarbonate at 37°C overnight, at an enzyme to protein ratio of 1:100. Peptides were collected by centrifugation, and the sample was acidified by addition of TFA to a final concentration 0.1% TFA. Samples were desalted using macrotrap (Peptide Macrotrap, Michrom Bioresources, Inc., CA, USA). Peptides were eluted by 200 μL 80% acetonitrile in 1% aqueous TFA. Eluted peptide samples were dried in SpeedVac Concentrator (Eppendorf, CR) and kept at -80°C until analyzed.

### LC-MS/MS analysis and data processing

Samples were solubilized in 10 μl of 60% (v/v) aqueous acetonitrile and sonicated for approx. 2 min. Next, 40 μl of 0.1% (v/v) aqueous TFA solution were added, and the samples were sonicated for another 3 min. Peptides were analyzed using nano UHPLC (Easy-nLC 1000; Thermo Fisher Scientific, Odense, Denmark) coupled to the quadrupole-Orbitrap mass analyzer (Q Exactive; Thermo Fisher Scientific, Bremen, Germany). The sample (1 μl) was loaded onto Thermo Scientific Acclaim EasySpray PepMap C18 RSLC column (internal diameter 75 um, length 50 cm, 2 μm particle size, 100 Å pore size) maintained at a constant temperature (40°C) and equilibrated with 5% (v/v) acetonitrile in 0.1% (v/v) aqueous formic acid (FA). Peptides were separated with a 180-minute linear gradient (5–35%) of acetonitrile in 0.1% (v/v) aqueous solution of formic acid, flow rate 250 nl/min. Total run time was 210 min. Each sample was run in quadruplicate.

Data dependent acquisition on the Q Exactive operated in positive mode. Peptide parent ions were detected in a high resolution full scan (mass range 350–1500 *m/z*, 70,000 resolving power setting (resolving power defined as a full peak width at half maximum height at *m/z* 200)). The instrument was set so that 10 most intense ions of every full spectrum, meeting specific threshold criteria (minimum intensity threshold 1.7 x 10^4^, charge state >1), should be selected for MS/MS. Peptides were isolated with an isolation window of 3 Da, fragmented (HCD fragmentation with NCE 27 collision energy setting), and the resulting fragment ions were detected (17,500 resolving power setting). Other settings: target value 3 x 10^6^ and 1 x 10^5^ for full scan and MS/MS scan, respectively; maximum ion time 50 ms and 120 ms for full scan and MS/MS scan, respectively. Following their fragmentation the precursors were put on an exclusion mass list for 30 seconds.

Data processing: Thermo Scientific Proteome Discoverer v. 1.4 (Thermo Fisher Scientific, Bremen, Germany) software package was used for protein identification and quantitation. The spectra were searched using Mascot (Matrix Science, London, UK) search engine against the human subset of SwissProt database with added contaminant protein sequences (20,249 sequences in total) with the following search settings: cleavage specificity–trypsin; max. 2 missed cleavage sites; precursor mass tolerance 10 ppm; fragment mass tolerance 20 mDa; carbamidomethylation of Cys residues (+57,021) set as a static modification; heavy Arg and Lys residues (+10,008 and +8,014) set as dynamic modifications; maximum 3 dynamic modifications per peptide allowed. The search results were validated with decoy database search strategy using Percolator [23].

Quantitative analysis was based on the area under curve (AUC) for extracted ion chromatograms (6 ppm mass tolerance) of the respective peptide precursors. Protein ratio was calculated as the median of peptide ratios. Only unique peptides were considered.

Proteins confidently identified in both forward and reverse analyses with at least 2 peptides (1377 proteins) were futher evaluated. To normalize for minor differences in protein loading during mixing of “light” and “heavy” cells, SILAC ratios were log normalized. For the semi-quantitative expression analysis only the proteins with with at least 3 SILAC pairs in each (Forward and Reverse) experiment were included. As differentially expressed we considered proteins showing a protein ratio change of at least 1.5-fold and having protein ratio variability lower or equal 40%.

The mass spectrometry proteomics data have been deposited to the ProteomeXchange Consortium via the PRIDE partner repository with the dataset identifier PXD002034 (http://www.ebi.ac.uk/pride/archive/projects/PXD002034).

### Western blotting

Cells were lysed in NHT buffer (140 mM NaCl, 10 mM HEPES pH 7.4, 1.5% Triton X-100) and centrifuged at 18,000 g to clear away debris. Protein concentration in resulting supernatants was determined by the Bradford assay. Lysates were combined with SDS loading buffer containing 2-mercaptoethanol, and boiled for 5 min. Quadruplicate samples (50 μg) were separated on Mini-PROTEAN TGX Stain-Free Precast Gels (Bio-Rad) in Tris-glycine-SDS buffer (Bio-Rad). Electrophoresis was performed at a constant voltage for 20 minutes at 300 V per gel until the dye front reached the gel bottom. Proteins were transferred onto 0.45 μm Immobilon-P PVDF membranes (Millipore) in Trans-Blot Turbo™ Transfer System semi-dry blotter (Bio-Rad) using pre-set transfer settings. Membranes were incubated in PBS (Sigma) containing 0.1 Tween-20 (Promega) and 5% non-fat milk for 30 minutes. GAPDH was used as a loading control. As primary antibodies anti-deoxycytidine kinase mouse monoclonal antibody (sc-81245, Santa Cruz Biotechnology) diluted 1:200, anti-Bcl-2 mouse monoclonal antibody (610539, BD Biosciences) diluted 1:1000, anti-Btk rabbit polyclonal antibody and anti-phospho-Btk (Y233) rabbit antibody (3533 and 5082, Cell Signaling Technologies) both diluted 1:1000, anti-phosphoserine aminotransferase rabbit polyclonal antibody (ab96136, Abcam) diluted 1:500, anti-SH-PTP1 rabbit polyclonal antibody (sc-287, Santa Cruz Biotechnology) diluted 1:100,000 and anti-GAPDH rabbit polyclonal antibody (G9545, Sigma) diluted 1:10,000 were used. After thorough washing in PBS with 0.1% Tween-20, secondary horseradish peroxidase-conjugated anti-mouse (sc-2005) or anti-rabbit (sc-2313, both from Santa Cruz Biotechnology) was added (diluted 1:10,000). The signal was detected using LumiGLO Reserve (KPL, Gaithersburg, MD, USA) or Western Blotting Luminol Reagent (Santa Cruz Biotechnology) and membranes were exposed to X-ray films (Kodak, Rochester, NY, USA) or visualised using ChemiDoc MP Imaging System.

### Flow cytometry analysis of CD marker expression

Cells were resuspended in 1% immunoglobulin/phosphate-buffered saline solution (IVIG/PBS) to prevent non specific staining. Cells were then labeled with anti-human CD20-APC monoclonal antibody, clone HI40a (EXBIO, Praha, Czech Republic) or anti-human CD38-PE-Cy7 monoclonal antibody, clone HIT2 (BIOLEGEND, San Diego, CA) on ice for 30 minutes. Flow cytometry was then perfomerd on FACS Aria IIu (488 nm; 50 mW orbis, 633 nm, cube coherent 24 mW laser and a 355 nm 5SDU 20 mW laser, BD Biosciences, San Jose, CA, USA). Data were evaluated using BD FACS Diva 6 software.

## Results and Discussion

### Establishment of fludarabine-resistant Mino/FR cells

**Fig 1:**
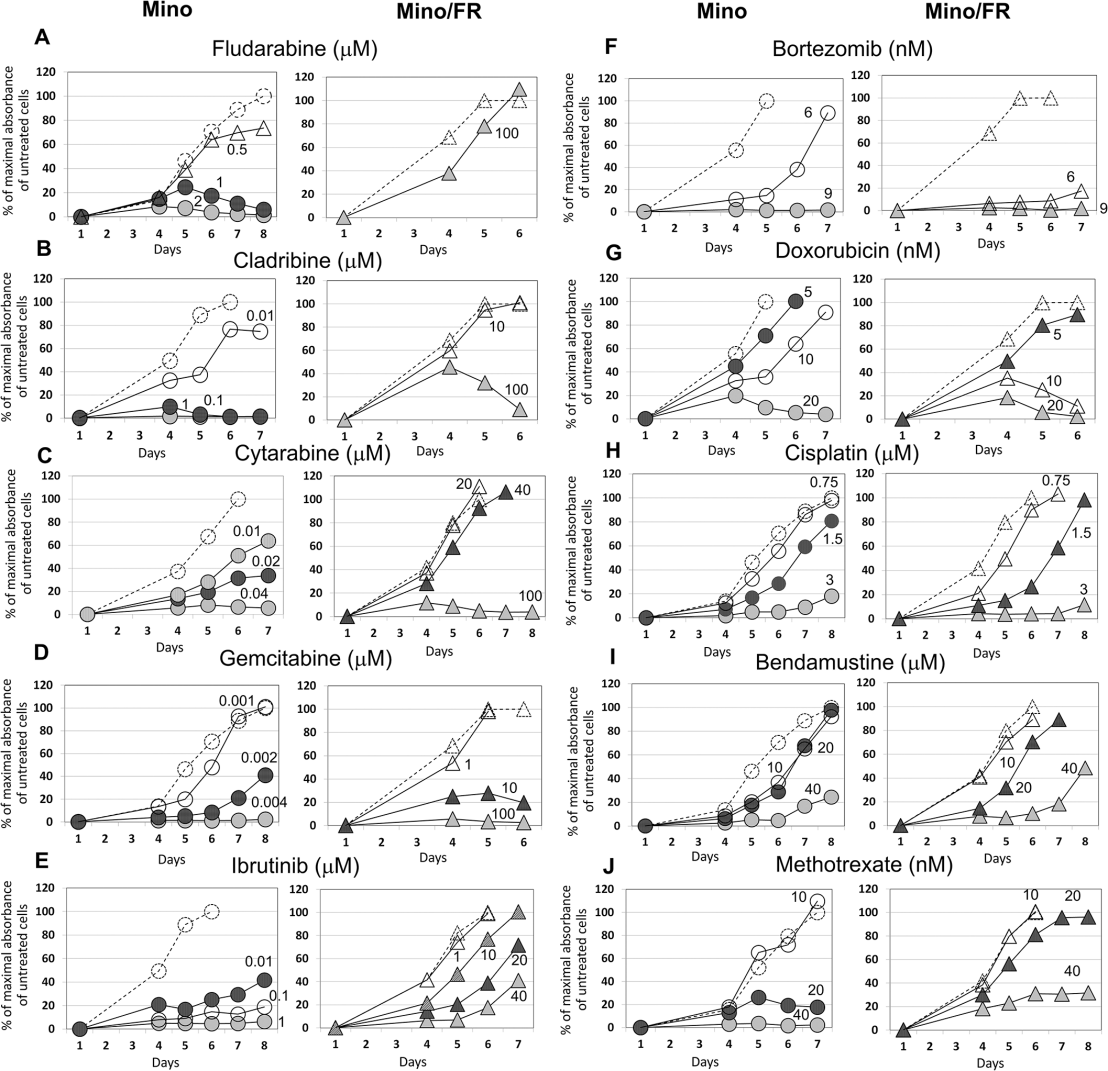
Proliferation of Mino and Mino/FR cells in presence of fludarabine and other anti-lymphoma agents. Cells were grown for 6–8 days in presence of increasing concentrations of **(A)** fludarabine, **(B)** cladribine, **(C)** cytarabine, **(D)** gemcitabine, **(E)** ibrutinib, **(F)** bortezomib, **(G)** doxorubicin, **(H)** cisplatin, **(I)** bendamustine and **(J)** methotrexate. Relative toxicity of the drugs was determined by the WST-8 cell proliferation assay, Dashed lines with open circles or triangles indicate cell proliferation in absence of an anti-lymphoma drug. Other curves represent the cells grown in increasing concentrations (indicated by the associated number) of the tested drug. Maximal absorbance (highest number of viable cells) of cells grown without an anti-lymphoma agent in each experiment was set as 100%. Standard deviations were < 5% for all measurements.

Mino cells represent an established model of MCL, derived from a Caucasian male patient [21]. Mino cells are sensitive to fludarabine treatment, with fludarabine LD_100_ approximatelly 1–2 μM. By growing Mino cells in the presence of increasing doses of fludrabine for a prolonged period of time we derived a fludarabine-resistant Mino subclone (Mino/FR). The resistant Mino/FR cells proliferated in 100 μM fludarabine. Toxicity of fludarabine in Mino and Mino/FR cells was determined *in vitro* by cell proliferation assay (Fig 1A).

### Mino/FR cells are cross-resistant to all tested purine and pyrimidine antinucleosides and to ibrutinib, but remain sensitive to other anti-cancer drugs

To characterize the Mino/FR cells in terms of their sensitivity to currently used anti-MCL agents we exposed the cells to a range of clinically used anti-lymphoma drugs with different mechanism of action. We included an anti-folate methotrexate, anti-nucleosides cytarabine, gemcitabine and cladribine, a DNA-intercalating agent doxorubicin, a DNA-modifier cisplatin, a proteasome-inhibitor bortezomib, a novel cytostatic with unique mechanism of anti-lymphoma activity bendamustine, and the recently approved taregted inhibitor of Bruton tyrosine kinase–ibrutinib. Mino and Mino/FR cells were exposed to the abovementioned drugs for 6–8 day. The appropriate range of drug concentrations was determined in preliminary experiments. Relative toxicity of the drugs was determined daily using WST-8 cell proliferation assay starting on the day 4 of the experiment. Triplicate samples (10,000 cells/well) were used for each drug concentration and measurement.

Mino/FR cells were significantly more resistant not only to fludarabine (proliferated in 100-fold higher concentrations compared to Mino cells and LD_100_ was not achieved) but also to another purine antinucleoside cladribine (LD_100_ approximately 1,000-fold higher), as well as to pyrimidine-derived cytarabine (LD_100_ approx. 2,000-fold higher) and gemcitabine (LD_100_ approx. 25,000-fold higher) (Fig 1B–1D). Moreover, Mino/FR cells were significantly more resistant to the BTK inhibitor ibrutinib (LD_100_ approx. 4,000-fold higher) (Fig 1E) and slightly more resistant to anti-folate MTX (LD_100_ at least 2-fold higher) (Fig 1J). On the other hand, Mino/FR cells were more sensitive to bortezomib and doxorubicin, tolerating only approximatelly 2-fold lower concentrations compared to Mino cells (Fig 1F and 1G). Sensitivity to alkylating agent bendamustine and cisplatin remained comparable to the original Mino cells (Fig 1H and 1I).

The marked cross-resistance of Mino/FR cells to purine and pyrimidine anti-nucleosides suggests a nucleoside-specific mechanism of resistance, while the observed resistence to BTK-inhibitor ibrutinib may suggest the deregulation of B-cell receptor (BCR) signaling in Mino/FR cells. The slightly increased sensitivity to an intercalating cytostatic doxorubicin suggested a potential deregulation of particular DNA repair mechanisms, while the slightly increased sensitivity to proteasome inhibitor bortezomib might suggest deregulation of NFB pathway. The data thus suggested multiple alterations in MCL cell homeostasis associated with the acquired resistance to fludarabine.

### Proteomic analysis of Mino versus Mino/FR cells

To uncover the processes responsible for and associated with the fludarabine resistance and to identifie the key molecules we performed in-depth proteomic analysis of Mino and Mino/FR cells.

**Fig 2:**
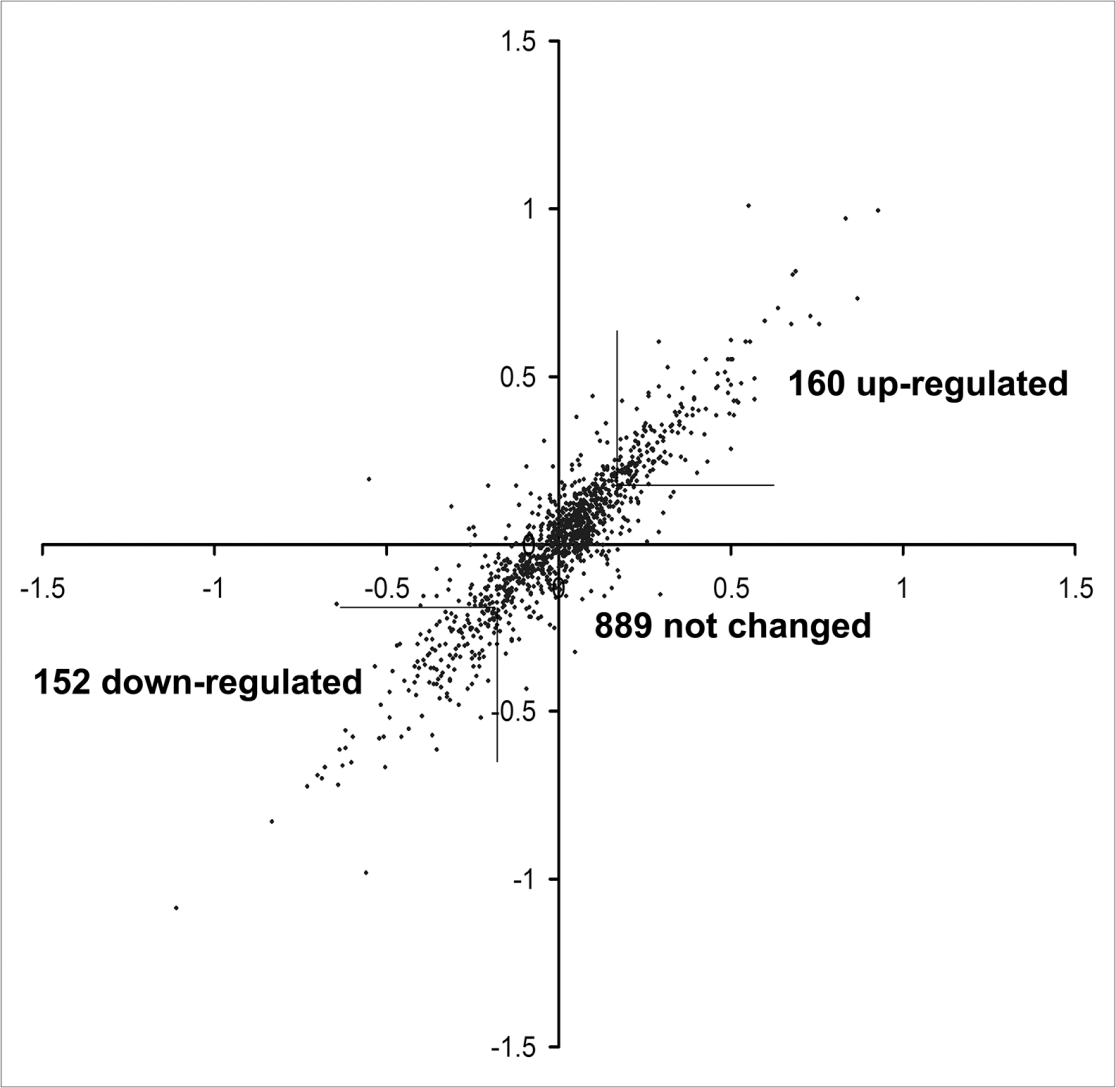
Correlation of protein expression ratios in forward and reverse SILAC experiments. Heavy/Light protein ratios (log values) from the forward experiment were plotted against log values of Light/Heavy protein ratios obtained from the reverse labeling experiment.

The differential proteomic analysis of Mino versus Mino/FR cells used metabolic incorporation of stable isotopes in cell culture (SILAC) [24]. Mino cells were first grown in the presence of ^13^C and ^15^N-labeled arginine and lysine while Mino/FR cells were grown in normal media with unlabeled amino acids (forward labeling). To make the results sufficiently robust, the experiment was repeated with swapped media (reverse labeling). Fig 2 shows the high correlation of expression ratios between forward and reverse experiments. We identified 1942 and 1700 proteins in forward and reverse experiment, respectively. Only the proteins identified in both analyses (1377 proteins) were further considered. For further quantitative analysis, we used 1201 proteins, each detected with at least 3 SILAC pairs and at the same time showing protein ration variability below 40 percent in both forward and reverse experiments. We identified 312 proteins in the resistant Mino/FR cells showing the fold change of at least 1.5, of which 152 were downregulated and 160 upregulated. (Table 1, for the full report see S1 Table).

**Table 1:** Differentially expressed proteins in Mino/FR cells identified by the proteomic analysis. Differentially expressed proteins with fold-change >1.5 are listed. Gene names are shown, proteins with fold-change >3 fold are present in bold letters).

**Proteins upregulated in Mino/FR cells**	
**DNA replication and repair**	FANCI, LIG1, MCM2, MCM3, MCM4, MCM5, MCM6, MCM7, MSH2, MSH6, NCAPD2, NCAPD3, NCAPG, NCAPH, PARP1, PDE12, RFC2, RFC3, **RFC4**, RFC5, RUVBL1, RUVBL2, SMC2, SMC4
**Purine and pyrimidine metabolism**	**ADA**, CTPS1, GMPS, HPRT1, IMPDH2, PNP, **RRM2**, UMPS, TYMS
**Aminoacyl tRNA biosynthesis**	CARS, DARS, GARS, EPRS, IARS, LARS, MARS, NARS, YARS
**Other processes**	ABCF1, ABCF3, **ACAD9**, ACLY, ACO2, ADRBK1, ALDH5A1, **ALOX5**, ANP32B, AP2M1, APEH, ATG3, ATP1A1, ATP1B3, ATP6V1C1, ATXN10, **BCL2**, **BLVRA,** CD2AP, CKAP5, CLPTM1L, COBRA1, CPNE1, **CPNE3**, CUL1, CYB5R3, **DCUN1D1**, DCXR, DDX17, DHCR24, DHCR7, **DHRS7B**, DHX30, DNAJC2, DNMT1, EEF1E1, EHD1, EIF2A, **EIF4A2**, EIF4E, EIF4G1, ERLIN1, ERO1L, ERP44, EXOSC7, **EZR**, FAM105B, FAM162A, FAM213A, FASN, FDFT1, FKBP5, **FXR1**, GAPVD1, GNA13, GNL1, GNL3, GOT1, **GRHPR**, GYG1, HEATR2, HMGB2, HNRNPD, HS2ST1, HSPA14, KHSRP, KIF11, KNTC1, KPNA2, **LBR**, LRMP, LSS, MYH10, NAA25, NAP1L4, NDUFAF4, NUP210, PA2G4, PAICS, PDCD6IP, PFKM, PFKP, **PHGDH**, PLCG2, PPM1G, PPP2R4, PRDX6, **PSAT1**, PSMD5, PSMG2, PTBP1, PTPRC, PTPRCAP, RAB7A, RRAS2, SBDS, SEC11C, SEC24A, SET, SFXN1, **SLC1A4**, SLC1A5, SLC25A1, SLC2A1, SRPR, ST13, **TPD52**, TRIP13, TROVE2, TUBA4A, TUBB4B, TUBGCP2, UBE2E1, VPRBP, XPNPEP1, XPO7, XPOT, ZW10
**Proteins Downregulated in Mino/FR cells**	
**Fatty acid metabolism**	ACAA2, **ACADVL, ACOT1** ACSL4, CPT2, HADHB
**Glutathione metabolism**	GSTK1, **GSTP1, G6PD**, IDH2, PGD
**CD molecules**	CD20 (MS4A1), CD38**, CD43, CD70, CD74**
**Adherens junctions**	CSNK2A2, MAPK1, PTPN1, **PTPN6**, SMAD3
**Other processes**	ALG5, ANXA2, APMAP, ARHGAP1, ARHGAP17, ARHGAP4, ARHGEF2, **ARL6IP5**, ARL8B, ASCC2, ATAD1, ATP2A3, ATPAF2, AUP1, BAX, **BCAP31**, BCAT2, **BTK**, CFA20, C1QBP, **CNDP2**, COPG2, CSTF3, CTPS2, DAGLB, **DCK**, DDX24, DDX3X, DKC1, DLST, DNAJA2, EIF4A1, EIF5A, ELMO1, EML4, FAM129C, FAM3C, FBXO7, FLAD1, **FLNA**, GBE1, GFPT1, GLOD4, GNPDA1, GOT2, HIST1H2AH, HIST1H2BK, HIST1H4A, HM13, HMGB3, HSP90B1, HSPH1, **ICAM1**, ICAM3, IER3IP1, IGF2BP3, INPP5D, IQSEC1, ITPR2, KPNA3, LTA4H, LRRFIP1, M6PR, MAT2A, ME2, MPDU1, MSN, MTA2, MYBBP1A, **NAGK**, NDUFA13, NDUFA9, NDUFB8, NDUFS1, NEK9, NLN, NRD1, NUDT19, MOB1B, OGDH, PDIA4, PEPD, PFAS, PGRMC1, POLR2B, POR, PPCS, PPIB, PRMT1,PRPF6, PRPSAP2, PSAP, PSME1, PSME2, PSMF1, PTPN2, PUS1, RASAL3, RNH1, **ROCK1,** RUFY1, SCRIB, SEC23IP, SEPT9, SLC25A5, SLC25A6, SLC38A5, SND1, SNRNP200, SRP54, STK4, TDP1, TMED4, TMX3, TOM1, TPM3, TPP2, TRMT6, TSR1, **TST, TSTA3**, UBLCP1, VARS, VAT1, VPS13C, VPS26A,VPS35, WDR1, XPO5, ZMPSTE24

### Bioinformatic analysis of the proteomic data

Functional annotation and pathway analysis of differentially expressed proteins using KEGG (Kyoto Encykopedia of Genes and Genomes) via DAVID (Database for Annotation, Visualization and Integrated Discovery) highlighted several processes affected by the fludarabine resistance in the Mino-FR cells. Most notably, it pointed toward the upregulation of proteins involved in the processes and pathways responsible for the DNA integrity maintenance. In particular, proteins ionvolved in DNA replication (p-val 1.1x10^-10^), mismatch repair (p-val 0.000001), nucleotide excision repair (p-val 0.005) and purine (p-val 0.03) and pyrimidine (p-val 0.06) metabolism were upregulated. In addition, proteins involved in aminoacyl t-RNA biosynthesis (p-val 1.6 x10^-7^) were also enriched among upregulated proteins.

Among the downregulated proteins the enrichment was less significant; proteins implicated in fatty acid metabolism (p-val 0.003), glutathione metabolism (p-val 0.006), and adherens junctions (p-val 0.03) were implicated.

### Verification of the landmark expression changes

**Fig 3:**
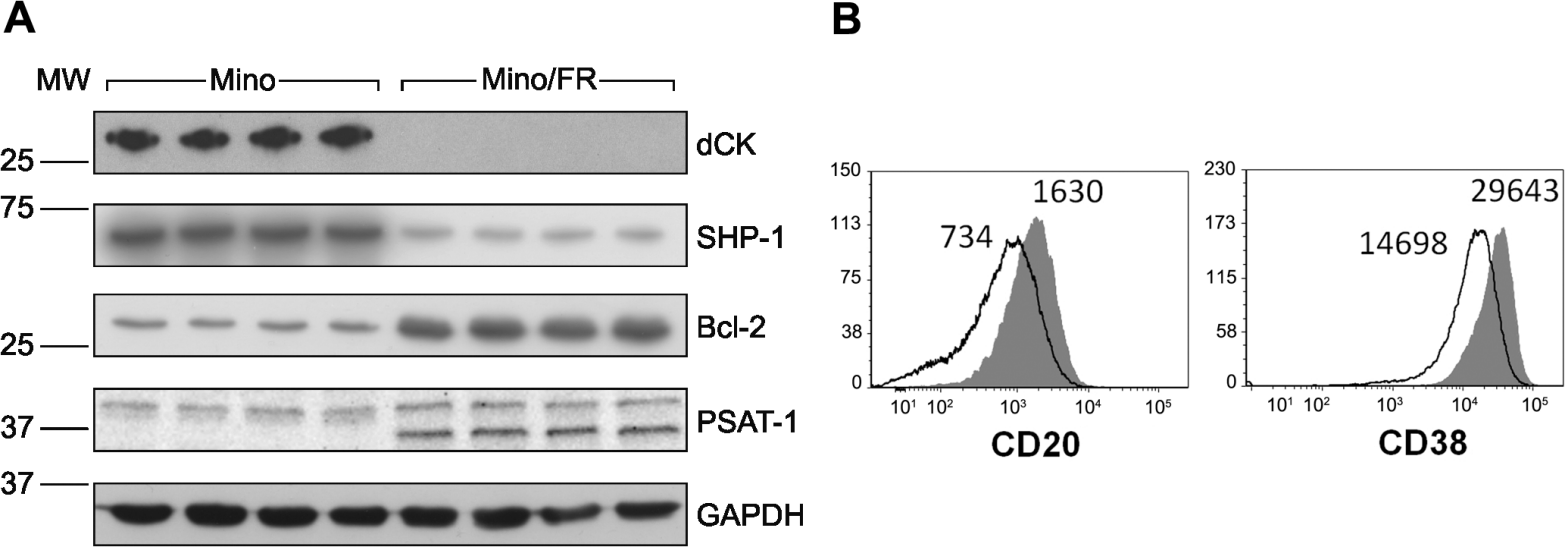
Verification of differential expression of the key proteins identified by proteomics. **(A)** Relative expression of four differentially expressed proteins—deoxycytidine kinase (dCK), phoshatase SHP-1 (alias PTPN6), Bcl-2 and phoshoserine aminotransferase (PSAT-1)–was determined by Western Blotting using specific antibodies in Mino and Mino/FR cells. GAPDH was used as a loading control. **(B)** Relative expression of two surface CD markers (CD20 and CD38) determined by flow cytometry using specific antibodies. Open histograms represent Mino/FR cells, full histograms show Mino cells. Histograms demonstrate approximately 2-fold decreased expression of CD20 and CD38 in Mino/FR cells as indicated by decreased median fluorescence intensity.

Among the proteins with the most pronounced (at least 5-fold) differential expression were these downregulated proteins: **deoxycytidine kinase (dCK)** and phosphatase **PTPN6** (alias SHP-1, Tyrosine-protein phosphatase non-receptor type 6), and these upregulated ones: **Bcl-2** and **phosphoserine aminotransferase** (PSAT). Using specific antibodies we confirmed the significantly altered expression of all four landmark proteins (Fig 3A). Observed downregulation of cell surface markers **CD20** (MS4A1) and **CD38** revealed by proteomics in Mino/FR cells was confirmed by flow cytometry using specific antibodies (Fig 3B).

### Deoxycitidine kinase, purine and pyrimidine utilization and metabolism

Several-fold downregulation of **deoxycytidine kinase (dCK)** was among the most prominent alterations in the fludarabine-resistant cells detected by the proteomic analysis. In fact, dCK was under detection limit in our Western blotting analysis in Mino/FR cells (Fig 2A), suggesting at least 10-fold downregulation, if not a total absence, of the protein. Deoxycytidine kinase is the key enzyme of deoxyribonucleoside salvage, a metabolic pathway that recycles products of DNA degradation and enables utilization of nucleosides from the environment. Deoxycytidine kinase is responsible for the first intracellular phosphorylation of deoxynucleosides (dCyd, dGuo and dAdo) including clinically relevant analogs cytarabine, cladribine, gemcitabine and fludarabine [9,25]. Despite its name, dCK is promiscuous and phosphorylates both pyrimidine and purine (anti)nucleosides [25]. Downregulation of dCK expression and activity has been demonstrated to cause antinucleoside resistance in human leukemic cell lines by others [8, 9, 26–28]. We have shown recently that the downregulation of dCK is responsible for the resistance to pyrimidine antimetabolite cytarabine in MCL cells, and for cross-resistance of the cytarabine-resistant cells to other antinucleosides [29]. Deoxycytidine kinase is the dominant, if not exclusive, kinase for fludarabine phosphorylation to its monophosphate [9,25–27]. We can thus reasonably conclude that the marked downregulation (or possibly even a total absence) of dCK is the critical alteration responsible for the fludarabine resistance in our MCL model. Substrate promiscuity of dCK explains the cross-resistance of Mino/FR cells to all other purine and pyrimidine antinucleosides included in our study.

### Purine and pyrimidine metabolism

Deoxyribonucleotide triphosphates (dNTPs) essential for DNA replication and repair can be produced either by the *de novo* pathway or by the nucleoside salvage pathway. Rapidly proliferating leukemia and lymphoma cells utilize nucleosides from the environment using the nucleotide salvage pathway [30]. Downregulation of dCK in the resistant Mino/FR cells limits fludarabine activation, preventing or limiting its toxic effect. Simultaneously, it also limits utilization of natural purine and pyrimidine nucleosides (*dCyd*, *dAdo*, *dGuo*) from the environment. Mammalian cells with negligible dCK activity thus become highly dependent on *de novo* nucleotide synthesis [30, 31].

This seems valid also in our model. In the Mino/FR cells we observed a modest, nevertheless, consistent upregulation of many components of purine and pyrimidine metabolism, namely those of *de novo* synthesis and consecutive interconversion reactions. Upregulated were **GMP synthase, CTP synthase 1, inosine-5´-monophosphate dehydrogenase, uridine-5´-monophosphate synthase, purine nucleoside phosphorylase, adenosine deaminase, purine nucleoside phosphorylase, thymidilate synthase, and ribonucleoide reductase subunit M2.**

Ribonucleotide reductase is the key enzyme of the *de novo* pathway for the production of deoxyribonucleosides for DNA synthesis from ribonucleosides. Ribonucleotide reductase activity is known to be directly inhibited by fludarabine [6–9] resulting in depleted dNTP pool. Upregulation of ribonucleotide reductase and its increased activity have been associated with fludarabine resistance in leukemia cells [9].

The concurrent upregulation of the purine and pyrimidine synthetic machinery suggests an increased demand for dNTPs. Resistant cells need to compensate for the loss of deoxynucloside utilization from the environment (normaly facilitated by dCK) by the *de novo* synthesis of both purines and pyrimidines, and their conversion to deoxynucleosides by ribonucleoside reductase.

### DNA replication and repair

Fludarabine incorporation into DNA ultimately activates DNA damage response resulting in apoptosis, unless the DNA damage is effectively and rapidly repaired. Increased efficiency of DNA repair is a general mechanism of resistance to DNA-targeting drugs. Mino/FR cells lacking dCK activity face, however, another challenge. Disruption of dNTP pools may cause the misincorporation of nucleotides into DNA resulting in a replication stress which further increases the demand for an effective DNA repair. It has been clearly demonstrated that inactivation of dCK in leukemia cells leads to replication stress and activates DNA-damage response [31].

In general, cells with damaged DNA might escape the damage-triggered apoptosis in so far as they can, at least partially, cope with the inhibited replication, and repair the DNA damage. In agreement with the results of KEGG pathway analysis, we observed upregulation of the large number of proteins participating in both of those processes. DNA repair of a damage in the form of nicks or double-strand breaks is initiated by **poly[ADP-ribose] polymerase 1 (PARP-1).** We found PARP-1 upregulated in Mino/FR cells. PARP-1 acts as a sensor that is thought to organize the damage site chromatin and/or serve as a scaffold for the subsequent recruitment of repair proteins. PARP-1 directly associates with condensin I protein complex, a conserved multiprotein multifunction assembly partaking in DNA replication and DNA repair [32,33]. We observed upregulation of all five subunits of **condensin I protein complex (condensin complex subunits 1, 2, 3 and structural maintenance of chromosomes proteins 2 and 4).** Similarly, upregulated **Fanconi anemia group I protein (FANCI)** is recruited to stalled replication forks at the sites of DNA damage and aids to the repair of double strand lesions [34]. Among other proteins known to contribute to DNA repair we identified the upregulated **DNA replication licensing factors MCM2, MCM3, MCM4 MCM5, MCM6 and MCM7, replication factor C subunits 2, 3, 4 and 5**, **pontin, reptin, and DNA ligase 1.**

The tight orchestration of replication stress response is mediated by ATM, ATR and checkpoint kinases. Recently, Nek9 kinase has been identified as a key component of the regulatory cascade [35]. **Nek9 kinase** was also found upregulated in Mino/FR cells.

The upregulation of numerous proteins participating in the maintenance of DNA integrity clearly indicates increased demand for DNA repair in Mino/FR cells. Such an upregulation may either contribute to the fludarabine resistance (despite the limited dCK activity, small amounts of fludarabine may still be incoroporated into DNA) and/or compensate for the higher incorporation error rate due to the deficiency of dNTPs caused by insufficient dCK activity [31].

### Avoiding apoptosis. Altered Bax /Bcl-2 ratio in Mino/FR

In addition to the processes directly responsible for the resistance (silenced expression of dCK) and the secondary/adaptive changes represented by the changes in nucleotide metabolism, replication and DNA repair, cells can avoid drug-induced apoptosis by the alteration of downstream processes, namely, signaling pathways leading to apoptosis. Apoptosis is regulated, among others, by the Bax/Bcl2 ratio [36, 37], where Bax promotes apoptosis by binding to and antagonizing the anti-apoptotic Bcl-2 protein. Upregulation of Bcl-2 as the mechanism of avoiding cell death has been documented in drug resistant cancer cells previously [38]. In our study, **Bcl-2** was also among the most strongly upregulated proteins in Mino/FR (Fig 3A), while expression of **Bax** was downregulated.

Markedly decreased Bax/Bcl-2 ratio in Mino/FR cells most likely reflects an anti-apoptotic process. This alteration *per se* does not, however, confer universal resistance to apoptosis as demonstrated by the preserved sensitivity of Mino/FR cells to cisplatin and bendamustine (Fig 1H and 1I). We hypothesize that the decreased Bax/Bcl-2 ratio might counterbalance the adverse alterations of the cell metabolism that result from the downregulation of dCK. Importantly, the Bcl-2 upregulation represents a potential therapeutic target for fludarabine-resistant MCL cells.

### High sensitivity of Mino/FR to Bcl-2 inhibitor ABT-199

**Fig 4:**
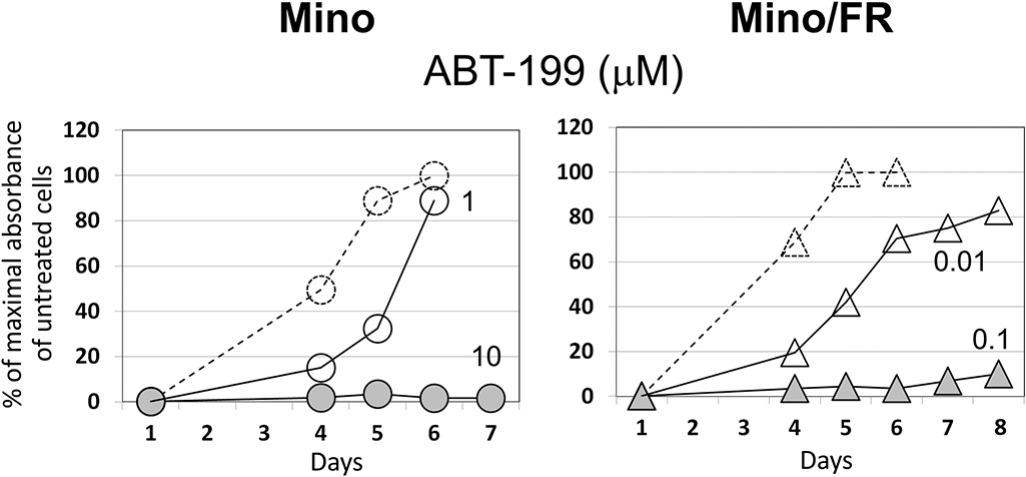
Mino/FR cell are highly sensitive to ABT-199. Proliferation of Mino and Mino/FR cells in presence of of 0.01–10 μM Bcl-2 inhibitor ABT199. Cells were grown for 6–8 days in presence ABT199. Relative toxicity of the drugs was determined by the WST-8 cell proliferation assay. Dashed curves and open circles or triangles indicate cell proliferation in absence of ABT199. Maximum absorbance (highest number of viable cells) of cells grown without ABT199 experiment was set as 100%. Other curves represent the cells grown in increasing concentrations (indicated by the associated number) of ABT199. Standard deviations were < 5% for all measurements.

Bcl-2 is an attractive therapeutic target. Sevreal Bcl-2 inhibitors have been developed and are currently in clinical tests. Highly selective Bcl-2 inhibitor ABT-199 showed great promise in a wide range of B-cell malignancies, with greatest efficacy reported in chronic lymphocytic leukemia and mantle cell lymphoma [39, 40]. In order to evaluate the potential of ABT-199 for the therapy of antinucleoside-resistant MCL, we tested relative toxicity of ABT-199 in Mino and Mino/FR cells. We observed markedly increased sensitivity of Mino/FR cells to ABT-199 compared to Mino cells (Fig 4). While Mino cells rapidly proliferated in 1μM ABT-199, proliferation of the Bcl-2 –overexpressing Mino/FR cells was reduced already at 0.01 μM ABT-199 while 0.1 μM ABT-199 effectively killed all Mino/FR cells in culture (LD_100_ 10-100-fold lower). To ensure, that the phenomenon is not exclusive to Mino/FR cells, we determined the relative expression of Bcl-2 and sensitivity to ABT-199 in another MCL cell line REC-1 and its cytrabine (CR) and fludarabine (FR) resistant subclone REC-1/CR/FR which we derived and characterized previously [29]. We demonstrated that the antinucleoside-resistant REC-1 cells also overexpress Bcl-2 and are more sensitive to ABT199 treatment *in vitro*. (S1 Fig). Our observation thus suggests high potential of the novel anti-Bcl-2 drug ABT-199 for the therapy of fludarabine-resitant MCL.

### B-cell receptor signalling. Bruton tyrosine kinase (BTK) and the resistance to ibrutinib

**Fig 5:**
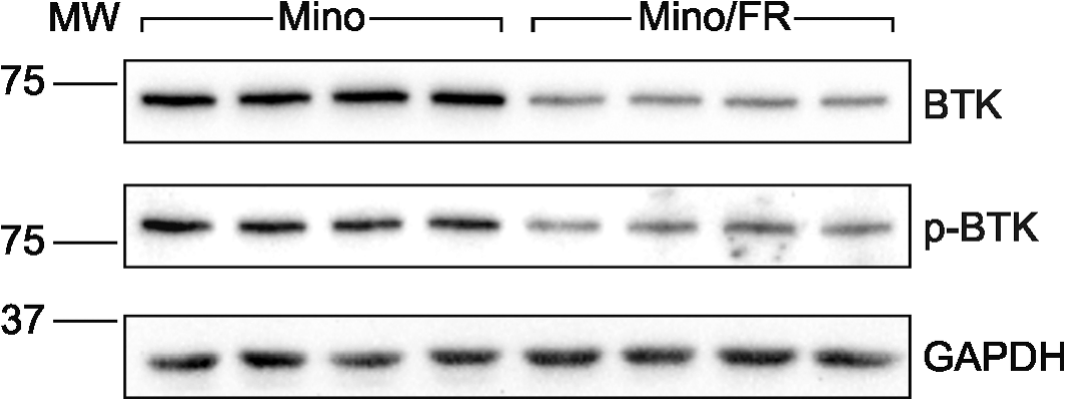
Decreased levels of total BTK and its activated p-BTKY^233^ form in Mino/FR cells. Relative expression of total and phosphorylated (active) p-BTK233 was determined by Western blotting using specific antibodies in Mino and Mino/FR cells. GAPDH was used as the loading control.

As we demonstrated in the initial battery of toxicity tests, fludarabine-resistant Mino/FR cells were significantly cross-resistant to ibrutinib, an inhibitor of Bruton tyrosine kinase (BTK)—recently approved for the therapy of relapsed/refractory mantle cell lymophoma [41]. BTK is a key component of B-cell receptor (BCR) signaling crucial to cell survival and proliferation during B-cell developement [42], and implicated in pathogenesis of B-cell malignancies, including MCL [3, 43]. Upon complex BCR stimulation, BTK is phosphorylated and the activated BTK phosphorylates phospholipase C gamma 2 (PLCG2). In turn, several downstream target pathways are activated including transcription factor NF-κB and mitogen-activated protein kinase (MAPK), leading to cell survival and proliferation [44]. Our proteomic analysis identified strong downregulation of **BTK** in Mino/FR. Because of its therapeutic importance, we verified both total and activated (phoshpo-BTK^Y233^) BTK levels in Mino and Mino/FR cells using Western blotting (Fig 5). BTK expression and p-BTK levels were markedly decreased in Mino/FR cells, clearly indicating the disruption of BCR signaling in Mino/FR cells. This provides the explanation for the observed resistance of Mino/FR cells to ibrutinib.

Still, many other players may contribute to the complex sum of signals that decide the cellular fate and response to ibrutinib. Among the downregulated proteins identified in Mino/FR cells there were two phosphatases known to contribute to BCR signalling as well—**phosphatase PTPN6 (alias SHP-1) and phosphatidylinositol 3,4,5-trisphosphate 5-phosphatase 1 (alias SHIP-1)** [45, 46]. We also observed downregulation and **MAPK kinase**, one of the downstream effectors of BCR signalling. Other molecules involved in different signalling cascades (such as **STAT3, kinases CSNK2A2 and ROCK, and phosphatases PTPN1 and PTPN2,** all downregulated in Mino/FR cells) could also contribute to Mino/FR survival via signalling crosstalk.

Nevertheless, the association of marked ibrutinib resistance with fludarabine resistance in Mino/FR cells, accompanied by the downregulation of BTK and p-BTK, are important observations indicating that disrupted BCR signalling may limit the effective usage of ibrutinib in MCL patients with fludarabine-resistant disease.

### Decreased expression of CD20 and other CD antigens, and the potential implications

Among the proteins downregulated in MinoFR cells we found six proteins belonging to the leukocyte CD (cluster of differentiation) antigens, namely, **CD20 (MS4A1), CD38, CD70, CD74 and CD43 (leukosialin).** Importantly, no CD antigen was identified as upregulated. Loss of the CD antigens in the resistant cells may be specific to resistance developement and/or reflect the shift of the resistant lymphoma cells toward differently matured B-cell phenotype.

Since most of the CD proteins are expressed on the cell surface, they represent attractive therapeutic targets. Downregulated expression of CD molecules may theoretically limit the efficacy of therapeutic antibodes such as rituximab (anti-CD20) used in lymphoma therapy. Recently, Kluin-Nelemans et al. published their landmark paper on the survival benefit of maintenance rituximab (R) in the elderly patients with newly diagnosed mantle cell lymphoma [2]. Interestingly, the influence of maintenance rituximab was detected only in patients who received R-CHOP (cyclophosphamide, vincristin, doxorubicin and prednisone), but not in those who received R-FC (fludarabine, cyclophosphamide). In the light of our own data, the downregulation of CD20 in those MCL cells that had survived fludarabine-based induction therapy might at least partially contribute to the failure of rituximab maintenance observed in that cohort of patients.

### Metabolic alterations in Mino/FR cells

Among the highly upregulated proteins in the resistant MCL cells were two enzymes responsible for two consecutive steps of serine synthesis, namely **D-3-phosphoglycerate dehydrogenase (PHGDH) and phosphoserine aminotransferase** (PSAT1, verified by Western blotting, Fig 3A). Since serine is an essential compound for the *de novo* nucleotide biosynthesis (as a source of carbon moieties in the folate cycle) the upregulation of serine production may therefore satisfy the increased demand for stimulated *de novo* nucleotide synthesis in Mino/FR cells, which are unable to recycle or obtain nucleosides from the environment due to the missing or defficient dCK activity. This hypothesis seems to be supported by the concomitant upregulation of **neutral amino acid transporter A (SLC1A4)** and **neutral amino acid transporter B (SLC1A5),** transporters specific for serine, alanine, cysteine, and threonine. Interestingly, upregulation of PSAT1 has been shown to increase proliferation and tumorigenic potential, as well as to protect tumor cells from oxaliplatin toxicity [46]. Whether the PSAT1 effect is directly connected with nucleotide metabolism or rather a more general compensatory metabolic alteration remains to be determined.

The downregulation of six enzymes partaking in the metabolism of fatty acids, (**carnitine O-palmitoyltransferase 2, trifunctional enzyme subunit beta, acyl-CoA thioesterase, 3-ketoacyl-CoA thiolase, long-chain-fatty-acid-CoA ligase 4, and very long chain specific acyl-CoA dehydrogenase**) accompanied by the downregulation of mitochondrial **ADP/ATP translocases 2 and 3** as well as other important mitochondrial proteins, may suggest a complex metabolic re-arrangement in Mino/FR cells.

Marked up-regulation (5-6-fold) of **biliverdin reductase** may contribute to the resistant phenotype. Biliverdin reductase converting biliverdin to bilirubin is a potent intracellular antioxidant, which is induced under hypoxia and contributes to hypoxia-induced resistance to doxycycline, paclitaxel and temozolomide in cancer cells [47, 48]. Whether biliverdin reductase contributes to antinucleoside resistance in our model or merely reflects increased oxidative stress remains to be determined. Similarly, upregulation of two key glutathione transferases, **GSTP1** and **GSTK1,** in Mino/FR may have several explanations. Upregulation of GSTP1 has been linked to an aquired drug resistance, including drugs which are not substrates for glutathionylation-based detoxification (e.g.antimetabolites) [49].

Interestingly, nine **aminoacyl tRNA synthases** were upregulated in the Mino/FR cells. In addition to their canonical roles, these molecules exert various other functions as recently reviewed [50]. Their in contribution to the survival of resistant cells remains to be elucidated.

## Conclusions

**Fig 6:**
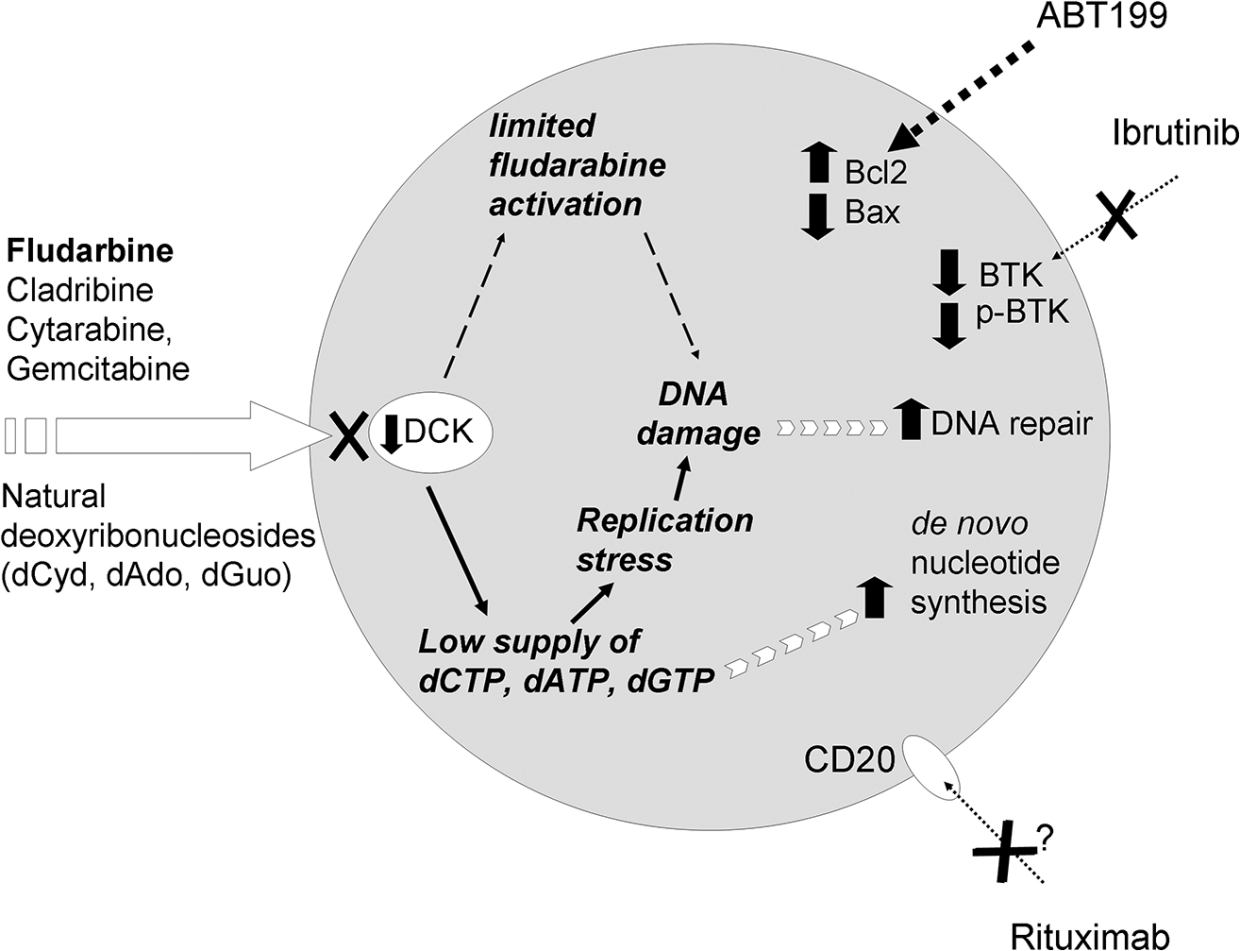
Schematic illustration of the processes associated with fludarabine resistance in Mino/FR cells summarizes the landmark observations in our analyses.

Detailed molecular analysis of therapy-resistant tumor cells is essential for in-depth understanding of causative mechanisms, as well as contributing and compensatory processes. Such knowledge then potentialy opens a way to the personalized therapy of the drug-resistant malignancy. Here we provided a detailed functional and proteomic snapshot of molecular mechanisms associated with the aquired fludarabine resistance of mantle cell lymphoma cells. Our data suggest the silencing of dCK as the probable causative mechanism of the resistance to fludarabine and of the cross-resistance to other antinucleotides, both pyrimidine- and purine-derived. In addition to the downregulation of dCK, we identified several secondary contributing or compensatory processes associated with the development of fludarabine resistance. Ineffective use of antinucleosides from the environment induces replicative stress and makes the resistant cells highly dependent on *de novo* nucleotide synthesis and effective DNA repair (Fig 6). Some of the identified alterations might have direct therapeutic consequences, including the downregulation of BTK (associated with the decreased sensitivity of fludarabine-resistant cells to ibrutinib) or the upregulation of Bcl-2 (responsible for an increased sensitivity to ABT-199). Our detailed functional and proteomic analysis of mantle cell lymphoma model of acquired resistance to fludarabine thus provides a proof-of-concept that might be exploited in the clinical setting (namely in mantle cell lymphoma and chronic lymphocytic leukemia) for prediction of optimal treatment strategies for those patients who fail fludarabine-based regimen. Due to the remarkable sensitivity and speed of high-resolution mass spectrometry, enabling rapid and detailed molecular characterization of small populations of cancer cells, proteomics may contribute to the formulation of individualized therapies in near future.

## Supporting Information

S1 Fig Bcl-2 expression and ABT199 toxicity in REC-1 and REC-1/CR/FR cells. Cytrabine- and fludarabine resistant subclone REC-1/CR/FR has been derived and characterized previously from an established MCL cell line REC-1 [29]. A) Expression of Bcl-2 in REC-1 and antinucleoside resiatnt REC-1/CR/FR cells. Relative expression of Bcl-2 was determined by Western blotting using specific antibodies in total cell lystates. GAPDH was used as the loading control. B) Proliferation of REC-1 and REC-1/CR/FR cells in presence of Bcl-2 inhibitro ABT199. Proliferation of REC-1 and REC-1/CR/FR cells in presence of of 0.01–10 μM Bcl-2 inhibitor ABT199 was determined. Cells were grown for 6–7 days in presence ABT199. Relative toxicity of the drugs was determined by the WST-8 cell proliferation assay. Dashed curves and open circles or triangles indicate cell proliferation in absence of ABT199. Maximum absorbance (highest number of viable cells) of cells grown without ABT199 experiment was set as 100%. Other curves represent the cells grown in increasing concentrations (indicated by the associated number) of ABT199. Standard deviations were < 5% for all measurements.
(TIF)

S1 Table The complete list of differentially expressed proteins. The list of differentially expressed proteins identified in Mino/FR cells by SILAC analysis. The proteins are ordered according the observed fold-change. Downregulated and upregulated proteins are shown in two separate tables. Number of unique and total peptides identified, number of SILAC pairs and normalized SILAC ratio are diplayed for each protein.
(PDF)
