# Supplementary figures and images for: Detailed Functional and Proteomic Characterization of Fludarabine Resistance in Mantle Cell Lymphoma Cells
Source: PLoS One. 2015 Aug 18;10(8):e0135314. doi: 10.1371/journal.pone.0135314 (PMC4540412; doi:10.1371/journal.pone.0135314)

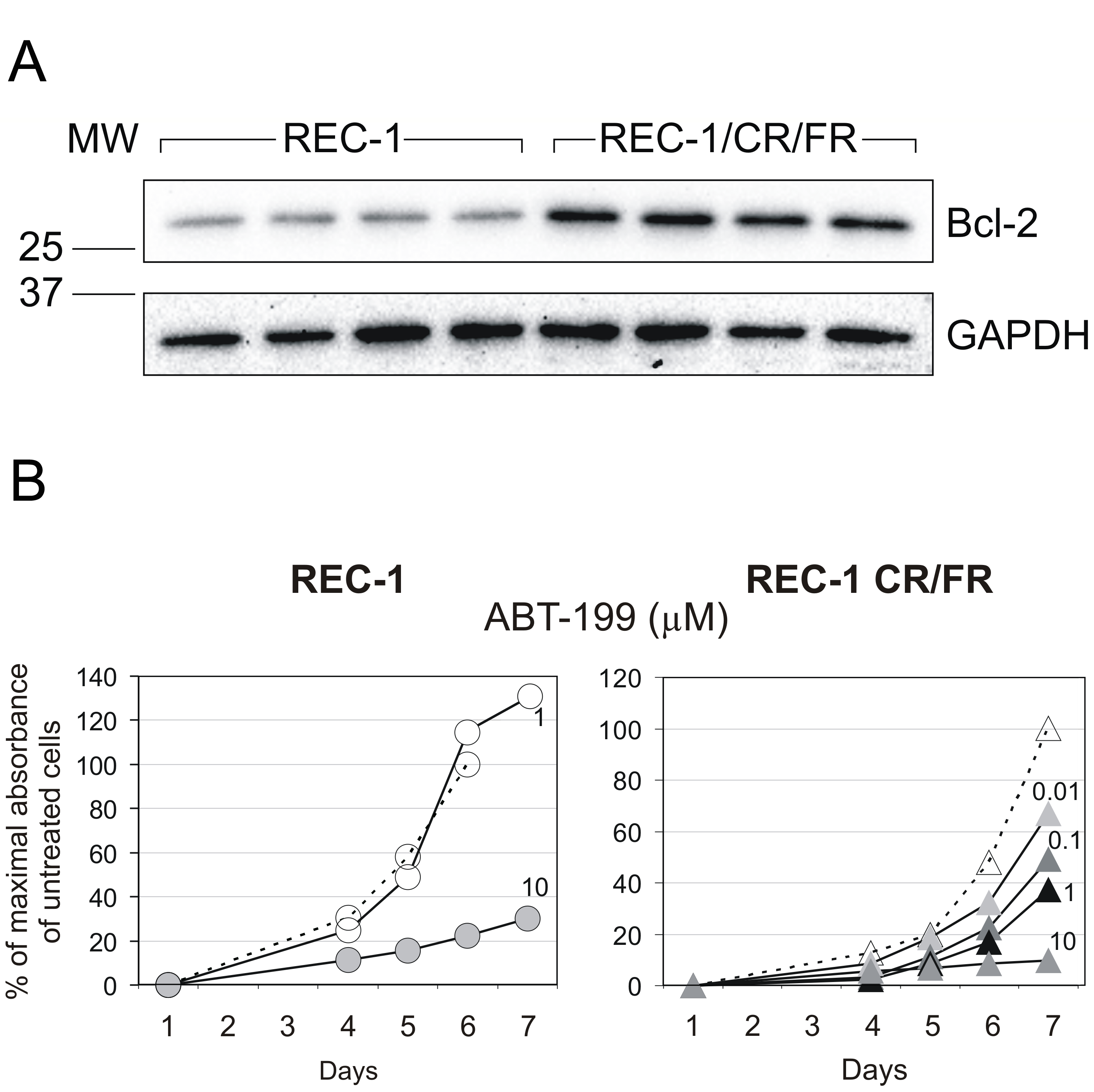

Supplement: S1 Fig — Cytrabine- and fludarabine resistant subclone REC-1/CR/FR has been derived and characterized previously from an established MCL cell line REC-1 [29]. A) Expression of Bcl-2 in REC-1 and antinucleoside resiatnt REC-1/CR/FR cells. Relative expression of Bcl-2 was determined by Western blotting using specific antibodies in total cell lystates. GAPDH was used as the loading control. B) Proliferation of REC-1 and REC-1/CR/FR cells in presence of Bcl-2 inhibitro ABT199. Proliferation of REC-1 and REC-1/CR/FR cells in presence of of 0.01–10 μM Bcl-2 inhibitor ABT199 was determined. Cells were grown for 6–7 days in presence ABT199. Relative toxicity of the drugs was determined by the WST-8 cell proliferation assay. Dashed curves and open circles or triangles indicate cell proliferation in absence of ABT199. Maximum absorbance (highest number of viable cells) of cells grown without ABT199 experiment was set as 100%. Other curves represent the cells grown in increasing concentrations (indicated by the associated number) of ABT199. Standard deviations were < 5% for all measurements. (TIF) [file pone.0135314.s001.tif]
