# Supplementary material for: Detailed Functional and Proteomic Characterization of Fludarabine Resistance in Mantle Cell Lymphoma Cells
Source: PLoS One. 2015 Aug 18;10(8):e0135314. doi: 10.1371/journal.pone.0135314 (PMC4540412; doi:10.1371/journal.pone.0135314)
Supplement: S1 Table — The list of differentially expressed proteins identified in Mino/FR cells by SILAC analysis. The proteins are ordered according the observed fold-change. Downregulated and upregulated proteins are shown in two separate tables. Number of unique and total peptides identified, number of SILAC pairs and normalized SILAC ratio are diplayed for each protein. (PDF) [file pone.0135314.s002.pdf]

PROTEINS DOWNREGULATED in Mino/FR

		FORWARD				REVERSE			
Accession	Description	# Unique Peptides	# Peptides	Heavy/Light Count	**Norm L/H ratio**	# Unique Peptides	# Peptides	Heavy/ Light Count	**Norm H/L ratio**
P29350	Tyrosine-protein phosphatase non-receptor type 6 [PTN6_HUMAN]	16	16	12	**0.03**	17	18	3	**0.14**
P27707	Deoxycytidine kinase [DCK_HUMAN]	4	4	3	**0.06**	2	2	3	**0.21**
Q86TX2	Acyl-coenzyme A thioesterase 1 [ACOT1_HUMAN]	5	5	5	**0.08**	5	5	6	**0.08**
P09211	Glutathione S-transferase P [GSTP1_HUMAN]	4	4	8	**0.15**	4	4	4	**0.15**
Q96KP4	Cytosolic non-specific dipeptidase [CNDP2_HUMAN]	15	15	26	**0.20**	13	13	26	**0.20**
P05362	Intercellular adhesion molecule 1 [ICAM1_HUMAN]	7	7	14	**0.21**	7	7	13	**0.20**
P51572	B-cell receptor-associated protein 31 [BAP31_HUMAN]	6	6	13	**0.21**	8	8	12	**0.22**
P49748	Very long-chain specific acyl-CoA dehydrogenase, mitochondrial [ACADV_HUMAN]	8	8	18	**0.23**	12	12	18	**0.19**
P21333	Filamin-A [FLNA_HUMAN]	41	45	64	**0.23**	58	64	119	**0.24**
Q13464	Rho-associated protein kinase 1 [ROCK1_HUMAN]	10	10	11	**0.24**	7	9	5	**0.25**
Q06187	Tyrosine-protein kinase BTK [BTK_HUMAN]	8	8	6	**0.24**	13	13	14	**0.28**
P11836	B-lymphocyte antigen CD20 [CD20_HUMAN]	5	5	14	**0.25**	7	7	23	**0.26**
Q9UJ70	N-acetyl-D-glucosamine kinase [NAGK_HUMAN]	4	4	3	**0.28**	5	5	5	**0.10**
P42765	3-ketoacyl-CoA thiolase, mitochondrial [THIM_HUMAN]	3	3	6	**0.29**	3	3	5	**0.43**
P32970	CD70 antigen [CD70_HUMAN]	2	2	11	**0.30**	3	3	14	**0.26**
O75915	PRA1 family protein 3 [PRAF3_HUMAN]	4	4	10	**0.30**	4	4	10	**0.33**
Q16762	Thiosulfate sulfurtransferase [THTR_HUMAN]	2	2	4	**0.31**	5	5	4	**0.27**
P04233	HLA class II histocompatibility antigen gamma chain [HG2A_HUMAN]	7	7	14	**0.31**	7	7	19	**0.22**
Q13630	GDP-L-fucose synthase [FCL_HUMAN]	2	2	5	**0.32**	3	3	9	**0.36**
P11413	Glucose-6-phosphate 1-dehydrogenase [G6PD_HUMAN]	17	17	58	**0.32**	19	19	58	**0.30**
O75352	Mannose-P-dolichol utilization defect 1 protein [MPU1_HUMAN]	2	2	7	**0.33**	2	2	9	**0.42**
P07602	Proactivator polypeptide [SAP_HUMAN]	2	2	3	**0.34**	2	2	3	**0.50**
P46926	Glucosamine-6-phosphate isomerase 1 [GNPI1_HUMAN]	3	3	5	**0.35**	4	4	7	**0.51**
Q9HDC9	Adipocyte plasma membrane-associated protein [APMAP_HUMAN]	7	7	13	**0.35**	7	7	13	**0.27**
Q93084	Sarcoplasmic/endoplasmic reticulum calcium ATPase 3 [AT2A3_HUMAN]	10	13	21	**0.36**	10	13	15	**0.39**
P14625	Endoplasmin [ENPL_HUMAN]	28	29	100	**0.37**	31	33	107	**0.37**
P13489	Ribonuclease inhibitor [RINI_HUMAN]	10	10	16	**0.37**	11	11	25	**0.28**
O15382	Branched-chain-amino-acid aminotransferase, mitochondrial [BCAT2_HUMAN]	3	3	5	**0.38**	4	4	7	**0.43**
Q92974	Rho guanine nucleotide exchange factor 2 [ARHG2_HUMAN]	6	6	14	**0.38**	6	6	10	**0.35**
Q9HAB8	Phosphopantothenate--cysteine ligase [PPCS_HUMAN]	2	2	4	**0.39**	4	4	4	**0.50**

P28331	NADH-ubiquinone oxidoreductase 75 kDa subunit, mitochondrial [NDUS1_HUMAN]	5	5	6	**0.39**	8	8	14	**0.41**
P62805	Histone H4 [H4_HUMAN]	5	5	13	**0.39**	6	6	26	**0.45**
Q96KK5	Histone H2A type 1-H [H2A1H_HUMAN]	2	4	8	**0.39**	2	4	12	**0.42**
Q8N5M1	ATP synthase mitochondrial F1 complex assembly factor 2 [ATPF2_HUMAN]	2	2	4	**0.40**	2	2	6	**0.65**
P16435	NADPH--cytochrome P450 reductase [NCPR_HUMAN]	7	7	12	**0.40**	11	11	17	**0.54**
Q07021	Complement component 1 Q subcomponent-binding protein, mitochondrial [C1QBP_HUMAN]	3	3	6	**0.40**	5	5	10	**0.31**
Q9Y606	tRNA pseudouridine synthase A, mitochondrial [TRUA_HUMAN]	4	4	3	**0.41**	5	5	11	**0.45**
Q92835	Phosphatidylinositol 3,4,5-trisphosphate 5-phosphatase 1 [SHIP1_HUMAN]	15	17	35	**0.41**	23	23	37	**0.44**
P23284	Peptidyl-prolyl cis-trans isomerase B [PPIB_HUMAN]	3	3	6	**0.41**	5	5	11	**0.52**
Q9UJA5	tRNA (adenine(58)-N(1))-methyltransferase non-catalytic subunit TRM6 [TRM6_HUMAN]	2	2	5	**0.41**	6	6	7	**0.47**
Q9NVJ2	ADP-ribosylation factor-like protein 8B [ARL8B_HUMAN]	4	4	8	**0.42**	5	5	9	**0.55**
O60814	Histone H2B type 1-K [H2B1K_HUMAN]	5	5	25	**0.42**	7	7	30	**0.47**
P28907	ADP-ribosyl cyclase 1 [CD38_HUMAN]	6	6	11	**0.42**	7	7	16	**0.39**
Q02218	2-oxoglutarate dehydrogenase, mitochondrial [ODO1_HUMAN]	17	17	29	**0.43**	14	14	25	**0.43**
Q9HC38	Glyoxalase domain-containing protein 4 [GLOD4_HUMAN]	2	2	7	**0.43**	5	5	10	**0.52**
O15347	High mobility group protein B3 [HMGB3_HUMAN]	3	3	5	**0.43**	5	5	15	**0.48**
Q2NL82	Pre-rRNA-processing protein TSR1 homolog [TSR1_HUMAN]	6	6	16	**0.43**	8	8	17	**0.38**
P18031	Tyrosine-protein phosphatase non-receptor type 1 [PTN1_HUMAN]	3	3	3	**0.43**	4	4	3	**0.43**
Q16795	NADH dehydrogenase [ubiquinone] 1 alpha subcomplex subunit 9, mitochondrial [NDUA9_HUMAN]	7	7	18	**0.44**	9	9	16	**0.48**
Q6DN90	IQ motif and SEC7 domain-containing protein 1 [IQEC1_HUMAN]	2	2	4	**0.44**	2	2	4	**0.38**
Q9Y6A4	UPF0468 protein C16orf80 [CP080_HUMAN]	2	2	5	**0.44**	2	2	7	**0.57**
Q06210	Glutamine--fructose-6-phosphate aminotransferase [isomerizing] 1 [GFPT1_HUMAN]	6	6	12	**0.44**	9	9	16	**0.42**
Q9HC35	Echinoderm microtubule-associated protein-like 4 [EMAL4_HUMAN]	7	7	12	**0.45**	9	9	15	**0.39**
O60256	Phosphoribosyl pyrophosphate synthase-associated protein 2 [KPRB_HUMAN]	2	2	7	**0.45**	5	6	10	**0.58**
P48735	Isocitrate dehydrogenase [NADP], mitochondrial [IDHP_HUMAN]	11	11	25	**0.45**	15	15	26	**0.35**
P19784	Casein kinase II subunit alpha' [CSK22_HUMAN]	4	5	8	**0.45**	4	5	9	**0.50**
P26038	Moesin [MOES_HUMAN]	14	21	48	**0.45**	20	31	76	**0.41**
O75083	WD repeat-containing protein 1 [WDR1_HUMAN]	7	7	18	**0.45**	10	10	20	**0.45**
Q9BQG0	Myb-binding protein 1A [MBB1A_HUMAN]	8	8	15	**0.45**	13	13	17	**0.37**
P12955	Xaa-Pro dipeptidase [PEPD_HUMAN]	3	3	7	**0.46**	4	4	9	**0.41**
Q8TD19	Serine/threonine-protein kinase Nek9 [NEK9_HUMAN]	10	10	21	**0.47**	10	10	19	**0.63**
P31153	S-adenosylmethionine synthase isoform type-2 [METK2_HUMAN]	6	6	12	**0.47**	9	9	18	**0.50**
O75436	Vacuolar protein sorting-associated protein 26A [VP26A_HUMAN]	3	3	9	**0.47**	5	5	15	**0.60**
Q13043	Serine/threonine-protein kinase 4 [STK4_HUMAN]	7	7	10	**0.47**	12	12	25	**0.36**
P07355	Annexin A2 [ANXA2_HUMAN]	10	10	31	**0.47**	13	13	27	**0.35**
Q96JJ7	Protein disulfide-isomerase TMX3 [TMX3_HUMAN]	7	7	6	**0.48**	3	3	4	**0.43**

Q9NUW8	Tyrosyl-DNA phosphodiesterase 1 [TYDP1_HUMAN]	2	2	6	**0.48**	5	5	8	**0.43**
Q14160	Protein scribble homolog [SCRIB_HUMAN]	4	4	5	**0.48**	7	7	3	**0.40**
O94906	Pre-mRNA-processing factor 6 [PRP6_HUMAN]	8	8	8	**0.48**	9	9	12	**0.47**
P36957	Dihydrolipoyllysine-residue succinyltransferase comp. of 2-oxoglutarate dehydrog. complex [ODO2_HUMAN]	6	6	11	**0.48**	5	5	13	**0.56**
Q8NBU5	ATPase family AAA domain-containing protein 1 [ATAD1_HUMAN]	3	3	6	**0.48**	3	3	6	**0.39**
A8MXV4	Nucleoside diphosphate-linked moiety X motif 19, mitochondrial [NUD19_HUMAN]	3	3	7	**0.49**	3	3	3	**0.62**
Q9Y6Y8	SEC23-interacting protein [S23IP_HUMAN]	6	6	17	**0.49**	10	10	21	**0.55**
O60884	DnaJ homolog subfamily A member 2 [DNJA2_HUMAN]	3	3	9	**0.49**	4	4	11	**0.45**
O15067	Phosphoribosylformylglycinamidine synthase [PUR4_HUMAN]	17	17	34	**0.49**	19	19	45	**0.56**
Q04446	1,4-alpha-glucan-branching enzyme [GLGB_HUMAN]	3	3	7	**0.50**	6	6	7	**0.58**
P60842	Eukaryotic initiation factor 4A-I [IF4A1_HUMAN]	7	16	23	**0.50**	8	18	17	**0.42**
Q86XR2	Niban-like protein 2 [NIBL2_HUMAN]	3	3	5	**0.50**	7	7	15	**0.47**
O95169	NADH dehydrogenase [ubiquinone] 1 beta subcomplex subunit 8, mitochondrial [NDUB8_HUMAN]	3	3	11	**0.50**	3	3	10	**0.49**
P28482	Mitogen-activated protein kinase 1 [MK01_HUMAN]	5	8	10	**0.50**	6	8	15	**0.61**
P32942	Intercellular adhesion molecule 3 [ICAM3_HUMAN]	4	4	13	**0.51**	5	5	9	**0.55**
O95831	Apoptosis-inducing factor 1, mitochondrial [AIFM1_HUMAN]	11	11	17	**0.51**	11	11	26	**0.55**
Q92520	Protein FAM3C [FAM3C_HUMAN]	5	5	9	**0.51**	4	4	5	**0.33**
P05141	ADP/ATP translocase 2 [ADT2_HUMAN]	3	13	16	**0.51**	3	13	13	**0.50**
P84022	Mothers against decapentaplegic homolog 3 [SMAD3_HUMAN]	2	2	4	**0.52**	1	2	3	**0.55**
O43847	Nardilysin [NRDC_HUMAN]	10	10	17	**0.52**	7	7	4	**0.46**
Q8NFF5	FAD synthase [FAD1_HUMAN]	5	5	12	**0.52**	8	8	11	**0.54**
P09960	Leukotriene A-4 hydrolase [LKHA4_HUMAN]	13	13	32	**0.52**	17	17	51	**0.55**
Q9Y679	Ancient ubiquitous protein 1 [AUP1_HUMAN]	2	2	3	**0.53**	2	2	9	**0.52**
Q9GZR7	ATP-dependent RNA helicase DDX24 [DDX24_HUMAN]	6	6	8	**0.53**	4	4	4	**0.50**
P52209	6-phosphogluconate dehydrogenase, decarboxylating [6PGD_HUMAN]	15	15	51	**0.53**	18	18	58	**0.57**
Q92530	Proteasome inhibitor PI31 subunit [PSMF1_HUMAN]	3	3	7	**0.54**	2	2	7	**0.50**
Q99873	Protein arginine N-methyltransferase 1 [ANM1_HUMAN]	7	7	18	**0.54**	10	10	35	**0.59**
P55084	Trifunctional enzyme subunit beta, mitochondrial [ECHB_HUMAN]	5	5	15	**0.55**	8	8	22	**0.61**
Q7L9L4	MOB kinase activator 1B [MOB1B_HUMAN]	4	4	10	**0.55**	4	4	9	**0.58**
P61011	Signal recognition particle 54 kDa protein [SRP54_HUMAN]	6	6	9	**0.55**	7	7	14	**0.67**
P29144	Tripeptidyl-peptidase 2 [TPP2_HUMAN]	18	18	35	**0.55**	18	18	35	**0.56**
P23368	NAD-dependent malic enzyme, mitochondrial [MAOM_HUMAN]	13	13	34	**0.55**	15	15	44	**0.55**
Q9P0J0	NADH dehydrogenase [ubiquinone] 1 alpha subcomplex subunit 13 [NDUAD_HUMAN]	2	2	6	**0.56**	4	4	8	**0.55**
Q9HAV4	Exportin-5 [XPO5_HUMAN]	10	11	18	**0.56**	16	16	24	**0.47**
O60784	Target of Myb protein 1 [TOM1_HUMAN]	3	3	3	**0.56**	2	2	3	**0.38**
Q14571	Inositol 1,4,5-trisphosphate receptor type 2 [ITPR2_HUMAN]	6	6	6	**0.56**	7	9	7	**0.46**

P12236	ADP/ATP translocase 3 [ADT3_HUMAN]	1	12	4	**0.56**	3	14	7	**0.56**
Q96T51	RUN and FYVE domain-containing protein 1 [RUFY1_HUMAN]	4	4	8	**0.56**	6	6	12	**0.53**
P17706	Tyrosine-protein phosphatase non-receptor type 2 [PTN2_HUMAN]	3	3	5	**0.57**	2	2	4	**0.43**
Q9Y673	Dolichyl-phosphate beta-glucosyltransferase [ALG5_HUMAN]	5	5	12	**0.57**	4	4	11	**0.66**
Q709C8	Vacuolar protein sorting-associated protein 13C [VP13C_HUMAN]	6	6	9	**0.57**	11	11	10	**0.46**
P06753	Tropomyosin alpha-3 chain [TPM3_HUMAN]	2	5	7	**0.58**	2	5	4	**0.40**
O00505	Importin subunit alpha-3 [IMA3_HUMAN]	5	5	12	**0.58**	3	5	6	**0.56**
Q8TCT9	Minor histocompatibility antigen H13 [HM13_HUMAN]	4	4	10	**0.58**	4	4	10	**0.55**
P98171	Rho GTPase-activating protein 4 [RHG04_HUMAN]	8	8	14	**0.59**	9	9	16	**0.47**
Q9Y5U9	Immediate early response 3-interacting protein 1 [IR3IP_HUMAN]	2	2	4	**0.59**	2	2	3	**0.45**
O60488	Long-chain-fatty-acid--CoA ligase 4 [ACSL4_HUMAN]	4	6	10	**0.59**	8	9	12	**0.43**
Q8NCG7	Sn1-specific diacylglycerol lipase beta [DGLB_HUMAN]	3	3	6	**0.59**	6	6	7	**0.60**
Q86YV0	RAS protein activator like-3 [RASL3_HUMAN]	4	4	7	**0.59**	6	6	9	**0.30**
O00264	Membrane-associated progesterone receptor component 1 [PGRC1_HUMAN]	3	4	9	**0.59**	3	5	8	**0.66**
Q9Y3I1	F-box only protein 7 [FBX7_HUMAN]	5	5	12	**0.59**	4	4	11	**0.52**
P63241	Eukaryotic translation initiation factor 5A-1 [IF5A1_HUMAN]	5	5	8	**0.60**	5	5	16	**0.66**
Q9NRF8	CTP synthase 2 [PYRG2_HUMAN]	5	5	15	**0.60**	9	9	20	**0.58**
Q9Y2Q3	Glutathione S-transferase kappa 1 [GSTK1_HUMAN]	2	2	4	**0.60**	3	3	8	**0.48**
Q8WVY7	Ubiquitin-like domain-containing CTD phosphatase 1 [UBCP1_HUMAN]	6	6	12	**0.60**	6	7	11	**0.62**
Q96QK1	Vacuolar protein sorting-associated protein 35 [VPS35_HUMAN]	7	7	15	**0.60**	10	10	20	**0.63**
Q9UL46	Proteasome activator complex subunit 2 [PSME2_HUMAN]	10	10	33	**0.60**	8	8	31	**0.62**
Q68EM7	Rho GTPase-activating protein 17 [RHG17_HUMAN]	5	5	6	**0.61**	5	5	3	**0.46**
P00505	Aspartate aminotransferase, mitochondrial [AATM_HUMAN]	13	13	29	**0.61**	12	12	44	**0.65**
P30876	DNA-directed RNA polymerase II subunit RPB2 [RPB2_HUMAN]	5	5	11	**0.61**	9	9	13	**0.58**
Q9UHD8	Septin-9 [SEPT9_HUMAN]	6	6	14	**0.62**	3	3	10	**0.63**
O75844	CAAX prenyl protease 1 homolog [FACE1_HUMAN]	6	6	14	**0.62**	3	3	5	**0.64**
Q99536	Synaptic vesicle membrane protein VAT-1 homolog [VAT1_HUMAN]	5	5	13	**0.62**	5	5	7	**0.39**
P16150	Leukosialin [LEUK_HUMAN]	3	3	10	**0.62**	4	4	10	**0.63**
Q07812	Apoptosis regulator BAX [BAX_HUMAN]	8	8	18	**0.62**	4	4	11	**0.57**
Q12996	Cleavage stimulation factor subunit 3 [CSTF3_HUMAN]	2	2	4	**0.63**	2	2	4	**0.57**
Q7Z7H5	Transmembrane emp24 domain-containing protein 4 [TMED4_HUMAN]	3	3	10	**0.63**	3	4	5	**0.66**
Q32MZ4	Leucine-rich repeat flightless-interacting protein 1 [LRRF1_HUMAN]	4	4	4	**0.63**	4	4	10	**0.50**
Q8WUX1	Sodium-coupled neutral amino acid transporter 5 [S38A5_HUMAN]	3	3	4	**0.63**	3	3	5	**0.59**
O00425	Insulin-like growth factor 2 mRNA-binding protein 3 [IF2B3_HUMAN]	3	3	5	**0.64**	9	9	17	**0.60**
P20645	Cation-dependent mannose-6-phosphate receptor [MPRD_HUMAN]	2	2	7	**0.64**	2	2	9	**0.67**
P13667	Protein disulfide-isomerase A4 [PDIA4_HUMAN]	16	16	52	**0.64**	18	18	54	**0.63**

Q9UBF2	Coatomer subunit gamma-2 [COPG2_HUMAN]	4	7	8	**0.64**	5	8	11	**0.62**
Q9BYT8	Neurolysin, mitochondrial [NEUL_HUMAN]	3	3	8	**0.65**	6	6	13	**0.54**
O60832	H/ACA ribonucleoprotein complex subunit 4 [DKC1_HUMAN]	5	5	4	**0.65**	4	4	7	**0.64**
P26640	Valine--tRNA ligase [SYVC_HUMAN]	23	23	65	**0.65**	34	34	79	**0.59**
Q06323	Proteasome activator complex subunit 1 [PSME1_HUMAN]	8	8	26	**0.65**	10	10	29	**0.60**
O00571	ATP-dependent RNA helicase DDX3X [DDX3X_HUMAN]	7	8	15	**0.65**	7	8	17	**0.60**
Q92556	Engulfment and cell motility protein 1 [ELMO1_HUMAN]	11	11	24	**0.65**	13	13	30	**0.62**
O75643	U5 small nuclear ribonucleoprotein 200 kDa helicase [U520_HUMAN]	30	30	61	**0.66**	34	34	69	**0.56**
Q9H1I8	Activating signal cointegrator 1 complex subunit 2 [ASCC2_HUMAN]	3	3	3	**0.66**	4	4	6	**0.61**
O94776	Metastasis-associated protein MTA2 [MTA2_HUMAN]	7	7	17	**0.66**	10	10	19	**0.50**
Q92598	Heat shock protein 105 kDa [HS105_HUMAN]	21	22	55	**0.66**	27	30	70	**0.57**
Q7KZF4	Staphylococcal nuclease domain-containing protein 1 [SND1_HUMAN]	23	23	73	**0.66**	28	28	79	**0.67**
P23786	Carnitine O-palmitoyltransferase 2, mitochondrial [CPT2_HUMAN]	4	4	7	**0.67**	6	6	13	**0.64**
Q07960	Rho GTPase-activating protein 1 [RHG01_HUMAN]	6	6	12	**0.67**	6	6	16	**0.61**

PROTEINS UP-REGULATED in Mino/FR

Accession	Description	FORWARD # Unique Peptides	FORWARD # Peptides	FORWARD Heavy/Light Count	FORWARD Norm L/H ratio	REVERSE # Unique Peptides	REVERSE # Peptides	REVERSE Heavy/Light Count	REVERSE Norm H/L ratio
Q9Y617	Phosphoserine aminotransferase [SERC_HUMAN]	2	2	3	**5.33**	5	5	3	**55.11**
P09917	Arachidonate 5-lipoxygenase [LOX5_HUMAN]	4	4	3	**3.55**	3	3	6	**10.18**
P10415	Apoptosis regulator Bcl-2 [BCL2_HUMAN]	3	3	9	**8.42**	3	3	4	**9.90**
Q14240	Eukaryotic initiation factor 4A-II [IF4A2_HUMAN]	4	13	13	**6.83**	3	13	3	**9.33**
P53004	Biliverdin reductase A [BIEA_HUMAN]	2	2	3	**4.78**	2	2	3	**6.36**
P51114	Fragile X mental retardation syndrome-related protein 1 [FXR1_HUMAN]	8	8	11	**7.39**	6	6	9	**5.41**
Q96GG9	DCN1-like protein 1 [DCNL1_HUMAN]	5	5	9	**4.32**	3	3	3	**5.03**
P55327	Tumor protein D52 [TPD52_HUMAN]	2	2	5	**5.38**	2	2	5	**4.79**
Q9NT62	Ubiquitin-like-conjugating enzyme ATG3 [ATG3_HUMAN]	5	5	12	**3.95**	3	3	7	**4.62**
P00813	Adenosine deaminase [ADA_HUMAN]	2	2	4	**5.70**	3	3	7	**4.54**
P31350	Ribonucleoside-diphosphate reductase subunit M2 [RIR2_HUMAN]	6	7	19	**4.71**	7	8	14	**4.52**
P43007	Neutral amino acid transporter A [SATT_HUMAN]	4	4	8	**3.15**	4	4	6	**4.05**
O75131	Copine-3 [CPNE3_HUMAN]	7	7	21	**3.61**	9	9	28	**4.02**
P62070	Ras-related protein R-Ras2 [RRAS2_HUMAN]	4	4	5	**3.49**	2	2	3	**4.01**
Q9H845	Acyl-CoA dehydrogenase family member 9, mitochondrial [ACAD9_HUMAN]	4	4	10	**3.10**	6	6	11	**3.57**
O43175	D-3-phosphoglycerate dehydrogenase [SERA_HUMAN]	12	12	28	**3.18**	14	14	39	**3.55**
Q9NUQ8	ATP-binding cassette sub-family F member 3 [ABCF3_HUMAN]	2	2	5	**2.68**	2	2	3	**3.55**
P15311	Ezrin [EZRI_HUMAN]	19	26	56	**3.21**	17	28	50	**3.54**
Q7Z4W1	L-xylulose reductase [DCXR_HUMAN]	4	4	6	**2.08**	3	3	6	**3.37**
Q14739	Lamin-B receptor [LBR_HUMAN]	7	7	18	**3.05**	9	9	36	**3.26**
Q9Y3A5	Ribosome maturation protein SBDS [SBDS_HUMAN]	4	4	5	**2.48**	4	4	3	**3.25**
P53007	Tricarboxylate transport protein, mitochondrial [TXTP_HUMAN]	7	7	20	**2.90**	5	5	14	**3.23**
Q6IAN0	Dehydrogenase/reductase SDR family member 7B [DRS7B_HUMAN]	2	2	4	**3.71**	2	2	6	**3.11**
Q9UBQ7	Glyoxylate reductase/hydroxypyruvate reductase [GRHPR_HUMAN]	2	2	5	**3.09**	3	3	6	**3.09**
P35249	Replication factor C subunit 4 [RFC4_HUMAN]	3	3	5	**3.39**	2	2	4	**3.01**
P35250	Replication factor C subunit 2 [RFC2_HUMAN]	6	6	19	**3.16**	5	5	15	**2.99**
Q9BRX8	Redox-regulatory protein FAM213A [F213A_HUMAN]	4	4	11	**1.96**	3	3	8	**2.97**
P35580	Myosin-10 [MYH10_HUMAN]	16	28	22	**2.91**	12	26	19	**2.95**
Q96A26	Protein FAM162A [F162A_HUMAN]	3	3	5	**2.29**	3	3	5	**2.93**

P00387	NADH-cytochrome b5 reductase 3 [NB5R3_HUMAN]	5	5	18	**2.86**	6	6	20	**2.92**
P52292	Importin subunit alpha-2 [IMA2_HUMAN]	7	7	14	**3.11**	6	6	14	**2.83**
Q7LGA3	Heparan sulfate 2-O-sulfotransferase 1 [HS2ST_HUMAN]	3	3	6	**1.82**	2	2	4	**2.83**
P42695	Condensin-2 complex subunit D3 [CNDD3_HUMAN]	3	3	6	**1.81**	3	3	5	**2.76**
Q9BVP2	Guanine nucleotide-binding protein-like 3 [GNL3_HUMAN]	4	4	7	**2.09**	4	4	9	**2.75**
P52701	DNA mismatch repair protein Msh6 [MSH6_HUMAN]	23	23	53	**2.47**	27	27	58	**2.73**
P52732	Kinesin-like protein KIF11 [KIF11_HUMAN]	15	15	28	**3.70**	10	10	17	**2.71**
Q9Y5K6	CD2-associated protein [CD2AP_HUMAN]	2	2	3	**2.46**	4	4	7	**2.69**
P21283	V-type proton ATPase subunit C 1 [VATC1_HUMAN]	4	4	7	**3.21**	3	3	6	**2.68**
P04818	Thymidylate synthase [TYSY_HUMAN]	4	4	10	**3.29**	3	3	9	**2.67**
Q15024	Exosome complex component RRP42 [EXOS7_HUMAN]	3	3	6	**1.52**	2	2	4	**2.66**
Q04637	Eukaryotic translation initiation factor 4 gamma 1 [IF4G1_HUMAN]	18	20	34	**3.31**	20	20	44	**2.65**
P11166	Solute carrier family 2, facilitated glucose transporter member 1 [GTR1_HUMAN]	4	4	10	**2.27**	4	4	10	**2.61**
P43246	DNA mismatch repair protein Msh2 [MSH2_HUMAN]	15	15	37	**2.28**	22	22	53	**2.55**
Q9UBM7	7-dehydrocholesterol reductase [DHCR7_HUMAN]	4	4	15	**1.68**	5	5	14	**2.52**
P08575	Receptor-type tyrosine-protein phosphatase C [PTPRC_HUMAN]	24	24	69	**2.45**	24	24	60	**2.52**
P40938	Replication factor C subunit 3 [RFC3_HUMAN]	5	5	7	**2.67**	5	5	7	**2.52**
P68366	Tubulin alpha-4A chain [TBA4A_HUMAN]	3	17	12	**2.55**	3	18	12	**2.50**
Q99798	Aconitate hydratase, mitochondrial [ACON_HUMAN]	7	7	14	**2.24**	5	5	4	**2.48**
Q9UBB4	Ataxin-10 [ATX10_HUMAN]	12	13	24	**2.17**	11	11	21	**2.46**
Q15758	Neutral amino acid transporter B(0) [AAAT_HUMAN]	7	7	13	**3.14**	6	6	12	**2.45**
Q7L2E3	Putative ATP-dependent RNA helicase DHX30 [DHX30_HUMAN]	6	6	6	**2.45**	8	8	15	**2.43**
Q16401	26S proteasome non-ATPase regulatory subunit 5 [PSMD5_HUMAN]	5	5	9	**2.36**	7	7	17	**2.43**
Q96BN8	Protein FAM105B [F105B_HUMAN]	7	7	9	**3.23**	3	3	6	**2.42**
P40937	Replication factor C subunit 5 [RFC5_HUMAN]	7	7	7	**2.87**	5	5	9	**2.42**
Q9NQW7	Xaa-Pro aminopeptidase 1 [XPP1_HUMAN]	11	11	29	**2.19**	11	11	22	**2.41**
P09874	Poly [ADP-ribose] polymerase 1 [PARP1_HUMAN]	22	22	52	**2.24**	23	23	68	**2.33**
P18858	DNA ligase 1 [DNLI1_HUMAN]	6	6	16	**1.67**	6	6	13	**2.28**
Q9Y265	RuvB-like 1 [RUVB1_HUMAN]	8	8	16	**2.12**	8	8	23	**2.27**
P50502	Hsc70-interacting protein [F10A1_HUMAN]	9	9	37	**2.00**	9	9	34	**2.26**
Q8WUM4	Programmed cell death 6-interacting protein [PDC6I_HUMAN]	16	16	32	**2.25**	17	17	32	**2.26**
P41252	Isoleucine--tRNA ligase, cytoplasmic [SYIC_HUMAN]	27	27	85	**2.12**	25	25	69	**2.26**
P51149	Ras-related protein Rab-7a [RAB7A_HUMAN]	10	10	20	**2.11**	8	8	26	**2.25**
Q92688	Acidic leucine-rich nuclear phosphoprotein 32 family member B [AN32B_HUMAN]	4	8	16	**1.85**	5	12	15	**2.25**
O95486	Protein transport protein Sec24A [SC24A_HUMAN]	4	4	11	**1.78**	6	7	17	**2.24**
Q9Y230	RuvB-like 2 [RUVB2_HUMAN]	9	9	23	**2.30**	11	11	29	**2.21**

Q8TEM1	Nuclear pore membrane glycoprotein 210 [PO210_HUMAN]	14	14	30	**1.86**	15	15	24	**2.20**
Q9BS26	Endoplasmic reticulum resident protein 44 [ERP44_HUMAN]	4	5	8	**1.77**	3	4	12	**2.20**
Q9BY44	Eukaryotic translation initiation factor 2A [EIF2A_HUMAN]	3	3	8	**2.14**	4	4	10	**2.18**
P13798	Acylamino-acid-releasing enzyme [ACPH_HUMAN]	9	9	27	**1.89**	10	10	32	**2.17**
Q13451	Peptidyl-prolyl cis-trans isomerase FKBP5 [FKBP5_HUMAN]	10	10	27	**2.03**	9	9	20	**2.17**
P51649	Succinate-semialdehyde dehydrogenase, mitochondrial [SSDH_HUMAN]	2	2	4	**1.78**	2	2	4	**2.16**
Q15645	Pachytene checkpoint protein 2 homolog [PCH2_HUMAN]	5	5	5	**1.67**	4	4	5	**2.13**
P08240	Signal recognition particle receptor subunit alpha [SRPR_HUMAN]	4	4	8	**2.65**	2	2	5	**2.13**
Q14344	Guanine nucleotide-binding protein subunit alpha-13 [GNA13_HUMAN]	4	5	3	**1.69**	4	5	5	**2.11**
Q9Y4B6	Protein VPRBP [VPRBP_HUMAN]	2	2	4	**1.97**	3	3	6	**2.10**
Q92945	Far upstream element-binding protein 2 [FUBP2_HUMAN]	8	9	19	**1.75**	9	11	23	**2.09**
O95347	Structural maintenance of chromosomes protein 2 [SMC2_HUMAN]	18	18	28	**2.01**	19	19	29	**2.07**
Q96HE7	ERO1-like protein alpha [ERO1A_HUMAN]	3	3	7	**1.58**	6	6	10	**2.07**
Q15257	Serine/threonine-protein phosphatase 2A activator [PTPA_HUMAN]	5	5	6	**1.52**	4	4	8	**2.05**
P53396	ATP-citrate synthase [ACLY_HUMAN]	31	31	96	**2.01**	36	36	94	**2.03**
Q9BY50	Signal peptidase complex catalytic subunit SEC11C [SC11C_HUMAN]	3	3	6	**1.66**	3	3	5	**2.02**
P06730	Eukaryotic translation initiation factor 4E [IF4E_HUMAN]	2	2	4	**1.94**	2	2	4	**2.01**
P49589	Cysteine--tRNA ligase, cytoplasmic [SYCC_HUMAN]	14	14	29	**1.70**	17	17	40	**2.00**
Q6L8Q7	2',5'-phosphodiesterase 12 [PDE12_HUMAN]	7	7	18	**1.70**	7	7	9	**1.99**
P48449	Lanosterol synthase [ERG7_HUMAN]	4	4	14	**1.83**	6	6	17	**1.99**
P00491	Purine nucleoside phosphorylase [PNPH_HUMAN]	8	8	25	**1.81**	9	9	21	**1.97**
P11172	Uridine 5'-monophosphate synthase [UMPS_HUMAN]	6	6	14	**1.85**	5	5	10	**1.93**
Q9NTJ3	Structural maintenance of chromosomes protein 4 [SMC4_HUMAN]	15	15	31	**1.85**	14	14	30	**1.92**
Q15392	Delta(24)-sterol reductase [DHC24_HUMAN]	3	3	6	**3.16**	4	4	6	**1.92**
P46976	Glycogenin-1 [GLYG_HUMAN]	4	4	9	**1.87**	2	2	4	**1.92**
P26583	High mobility group protein B2 [HMGB2_HUMAN]	3	4	13	**1.50**	4	8	17	**1.92**
P30041	Peroxiredoxin-6 [PRDX6_HUMAN]	12	12	36	**1.88**	14	14	41	**1.91**
P54577	Tyrosine--tRNA ligase, cytoplasmic [SYYC_HUMAN]	27	27	72	**1.73**	30	30	87	**1.90**
Q14761	Protein tyrosine phosphatase receptor type C-associated protein [PTCA_HUMAN]	4	4	9	**1.63**	2	2	6	**1.90**
Q9BPX3	Condensin complex subunit 3 [CND3_HUMAN]	7	7	21	**2.02**	9	9	27	**1.88**
Q8WX92	Negative elongation factor B [NELFB_HUMAN]	4	4	5	**1.81**	4	4	6	**1.87**
P41250	Glycine--tRNA ligase [SYG_HUMAN]	20	20	57	**1.73**	25	26	93	**1.85**
Q15003	Condensin complex subunit 2 [CND2_HUMAN]	3	3	3	**2.11**	5	5	7	**1.83**
Q96CW1	AP-2 complex subunit mu [AP2M1_HUMAN]	5	5	13	**1.97**	5	5	4	**1.82**
Q9H9B4	Sideroflexin-1 [SFXN1_HUMAN]	7	7	21	**1.64**	11	11	20	**1.82**
Q01105	Protein SET [SET_HUMAN]	5	5	17	**1.52**	6	6	16	**1.81**

Q15021	Condensin complex subunit 1 [CND1_HUMAN]	17	17	40	**2.27**	23	23	51	**1.81**
P68371	Tubulin beta-4B chain [TBB4B_HUMAN]	3	17	12	**1.71**	3	17	20	**1.81**
O75477	Erlin-1 [ERLN1_HUMAN]	2	4	3	**1.96**	2	3	4	**1.80**
Q96KA5	Cleft lip and palate transmembrane protein 1-like protein [CLP1L_HUMAN]	2	2	6	**2.33**	3	3	4	**1.78**
P10155	60 kDa SS-A/Ro ribonucleoprotein [RO60_HUMAN]	8	8	17	**1.81**	9	9	23	**1.77**
P54709	Sodium/potassium-transporting ATPase subunit beta-3 [AT1B3_HUMAN]	4	4	13	**1.72**	7	7	21	**1.77**
Q9H4M9	EH domain-containing protein 1 [EHD1_HUMAN]	20	23	76	**1.87**	21	23	76	**1.77**
P50748	Kinetochore-associated protein 1 [KNTC1_HUMAN]	6	6	6	**2.70**	4	4	7	**1.76**
P26358	DNA (cytosine-5)-methyltransferase 1 [DNMT1_HUMAN]	7	7	15	**2.01**	10	10	21	**1.76**
P51965	Ubiquitin-conjugating enzyme E2 E1 [UB2E1_HUMAN]	2	2	7	**1.89**	2	2	6	**1.76**
P36915	Guanine nucleotide-binding protein-like 1 [GNL1_HUMAN]	5	5	14	**1.87**	8	8	19	**1.75**
Q12912	Lymphoid-restricted membrane protein [LRMP_HUMAN]	10	10	24	**1.63**	9	9	20	**1.75**
P05023	Sodium/potassium-transporting ATPase subunit alpha-1 [AT1A1_HUMAN]	27	27	67	**1.65**	20	20	58	**1.75**
P49736	DNA replication licensing factor MCM2 [MCM2_HUMAN]	20	20	71	**1.69**	22	22	84	**1.74**
P14868	Aspartate--tRNA ligase, cytoplasmic [SYDC_HUMAN]	18	18	48	**1.62**	20	20	65	**1.74**
Q9NVI1	Fanconi anemia group I protein [FANCI_HUMAN]	6	6	11	**2.05**	4	5	5	**1.74**
Q969U7	Proteasome assembly chaperone 2 [PSMG2_HUMAN]	4	4	8	**1.88**	4	4	8	**1.74**
P12268	Inosine-5'-monophosphate dehydrogenase 2 [IMDH2_HUMAN]	4	4	10	**1.60**	6	6	14	**1.73**
Q14566	DNA replication licensing factor MCM6 [MCM6_HUMAN]	24	24	63	**1.62**	22	22	64	**1.73**
O43592	Exportin-T [XPOT_HUMAN]	14	14	42	**1.52**	13	13	29	**1.72**
O43264	Centromere/kinetochore protein zw10 homolog [ZW10_HUMAN]	4	4	5	**1.69**	4	4	9	**1.72**
P33991	DNA replication licensing factor MCM4 [MCM4_HUMAN]	17	17	40	**1.68**	17	17	33	**1.72**
Q92841	Probable ATP-dependent RNA helicase DDX17 [DDX17_HUMAN]	7	10	12	**1.62**	9	14	22	**1.72**
Q01813	6-phosphofructokinase type C [K6PP_HUMAN]	8	10	23	**1.66**	13	15	19	**1.71**
P08237	6-phosphofructokinase, muscle type [K6PF_HUMAN]	5	7	14	**1.58**	7	10	19	**1.71**
Q14008	Cytoskeleton-associated protein 5 [CKAP5_HUMAN]	19	20	51	**1.77**	23	24	53	**1.70**
P16885	1-phosphatidylinositol 4,5-bisphosphate phosphodiesterase gamma-2 [PLCG2_HUMAN]	29	30	86	**1.56**	28	30	80	**1.68**
P33992	DNA replication licensing factor MCM5 [MCM5_HUMAN]	27	27	92	**1.59**	29	29	95	**1.68**
Q9UIA9	Exportin-7 [XPO7_HUMAN]	12	12	15	**1.59**	14	14	22	**1.67**
P22234	Multifunctional protein ADE2 [PUR6_HUMAN]	12	12	42	**1.53**	14	14	43	**1.66**
Q8NE71	ATP-binding cassette sub-family F member 1 [ABCF1_HUMAN]	10	10	26	**1.88**	12	12	29	**1.66**
P56192	Methionine--tRNA ligase, cytoplasmic [SYMC_HUMAN]	19	19	41	**1.68**	22	22	49	**1.65**
O43776	Asparagine--tRNA ligase, cytoplasmic [SYNC_HUMAN]	14	14	50	**1.57**	15	15	56	**1.65**
P17812	CTP synthase 1 [PYRG1_HUMAN]	11	11	24	**1.85**	9	9	18	**1.65**
Q0VDF9	Heat shock 70 kDa protein 14 [HSP7E_HUMAN]	2	2	7	**1.61**	2	2	5	**1.64**
P26599	Polypyrimidine tract-binding protein 1 [PTBP1_HUMAN]	8	8	16	**1.53**	9	9	19	**1.64**

Q14103	Heterogeneous nuclear ribonucleoprotein D0 [HNRPD_HUMAN]	6	7	15	**1.50**	6	7	14	**1.64**
O15355	Protein phosphatase 1G [PPM1G_HUMAN]	8	8	22	**1.56**	11	11	30	**1.64**
Q14C86	GTPase-activating protein and VPS9 domain-containing protein 1 [GAPD1_HUMAN]	3	3	5	**2.51**	4	4	7	**1.64**
Q9UQ80	Proliferation-associated protein 2G4 [PA2G4_HUMAN]	13	13	35	**1.56**	15	15	60	**1.64**
Q99829	Copine-1 [CPNE1_HUMAN]	4	4	11	**1.52**	6	6	13	**1.63**
P07814	Bifunctional glutamate/proline--tRNA ligase [SYEP_HUMAN]	31	32	95	**1.61**	37	38	98	**1.63**
P25205	DNA replication licensing factor MCM3 [MCM3_HUMAN]	23	23	64	**1.58**	27	27	59	**1.62**
P33993	DNA replication licensing factor MCM7 [MCM7_HUMAN]	26	26	68	**1.60**	26	26	75	**1.62**
Q99543	DnaJ homolog subfamily C member 2 [DNJC2_HUMAN]	5	5	7	**1.67**	7	7	9	**1.60**
P37268	Squalene synthase [FDFT_HUMAN]	6	6	17	**1.80**	3	3	6	**1.60**
O43324	Eukaryotic translation elongation factor 1 epsilon-1 [MCA3_HUMAN]	3	3	8	**1.65**	3	3	3	**1.59**
P17174	Aspartate aminotransferase, cytoplasmic [AATC_HUMAN]	11	11	35	**1.64**	11	11	32	**1.57**
P49915	GMP synthase [glutamine-hydrolyzing] [GUAA_HUMAN]	10	10	20	**1.58**	9	9	15	**1.55**
Q14CX7	N-alpha-acetyltransferase 25, NatB auxiliary subunit [NAA25_HUMAN]	4	4	7	**1.69**	8	8	15	**1.55**
Q13616	Cullin-1 [CUL1_HUMAN]	7	7	15	**1.63**	7	7	7	**1.55**
Q86Y56	HEAT repeat-containing protein 2 [HEAT2_HUMAN]	5	5	9	**1.58**	8	8	19	**1.53**
Q99733	Nucleosome assembly protein 1-like 4 [NP1L4_HUMAN]	7	8	15	**1.53**	8	9	22	**1.52**
P49327	Fatty acid synthase [FAS_HUMAN]	59	61	207	**1.60**	69	71	242	**1.51**
Q9BSJ2	Gamma-tubulin complex component 2 [GCP2_HUMAN]	5	5	9	**1.83**	5	5	7	**1.51**
Q9P2J5	Leucine--tRNA ligase, cytoplasmic [SYLC_HUMAN]	30	30	75	**1.61**	35	35	90	**1.51**
P00492	Hypoxanthine-guanine phosphoribosyltransferase [HPRT_HUMAN]	5	5	21	**1.49**	6	6	18	**1.50**
P25098	Beta-adrenergic receptor kinase 1 [ARBK1_HUMAN]	5	5	9	**1.66**	10	10	24	**1.49**
Q9P032	NADH dehydrogenase [ubiquinone] 1 alpha subcomplex assembly factor 4 [NDUF4_HUMAN]	2	2	3	**1.65**	4	4	6	**1.48**
